# *TWF2* Drives Tumor Progression and Sunitinib Resistance in Renal Cell Carcinoma through Hippo Signaling Suppression

**DOI:** 10.1002/advs.202506367

**Published:** 2025-09-15

**Authors:** Liangmin Fu, Wuyuan Liao, Youyan Tan, Hansen Lin, Kun Ye, Xinwei Zhou, Mingjie Lin, Kangbo Huang, Minyu Chen, Jietao Wei, Haoqian Feng, Yuhang Chen, Jinwei Chen, Bohong Guan, Shan Li, Zhengkun Zhang, Anze Yu, Zihao Feng, Lizhen Zhang, Guannan Shu, Jun Lu, Wei Chen, Yihui Pan, Jiefeng Yang, Junhang Luo, Li Luo

**Affiliations:** ^1^ Department of Urology The First Affiliated Hospital of Sun Yat‐sen University Guangzhou Guangdong 518880 China; ^2^ Digestive Diseases Center Guangdong Provincial Key Laboratory of Digestive Cancer Research The Seventh Affiliated Hospital Sun Yat‐Sen University Shenzhen 528406 China; ^3^ Department of Urology The Second Xiangya Hospital of Central South University Changsha Hunan 410011 China; ^4^ Department of Urology Cancer Hospital Chinese Academy of Medical Sciences Shenzhen Center Shenzhen 518116 China; ^5^ Department of Urology Sun Yat‐sen University Cancer Center Guangzhou 510060 China; ^6^ State Key Laboratory of Oncology in South China Guangdong Provincial Clinical Research Center for Cancer Sun Yat‐sen University Cancer Center Guangzhou 510060 China; ^7^ Department of Urology Children's Hospital of Chongqing Medical University Chongqing 400014 China; ^8^ Department of Urology Guangzhou Women and Children's Medical Center Guangzhou Institute of Pediatrics Guangdong Provincial Clinical Research Center for Child Health Guangzhou Medical University Guangzhou 510623 China; ^9^ Department of Urology the Third Affiliated Hospital of Soochow University Changzhou 213000 China; ^10^ Department of Oncology The First Affiliated Hospital of Sun Yat‐sen University Guangzhou Guangdong 510080 China

**Keywords:** Hippo signaling, renal cell carcinoma, sunitinib resistance, tumor progression, *TWF2*

## Abstract

Renal cell carcinoma (RCC) remains a formidable clinical challenge, characterized by a high propensity for metastasis and the frequent emergence of intrinsic or acquired resistance to targeted therapies. However, the molecular mechanisms underlying sunitinib resistance and tumor progression in RCC are not fully understood. This study aims to identify Twinfilin actin‐binding protein (*TWF2*) as a key mediator of tumor aggressiveness and therapeutic resistance. *TWF2* expression is markedly upregulated in RCC cells, particularly in sunitinib‐resistant subtypes, and significantly associated with poor prognosis and therapeutic nonresponsiveness. Functional analyses demonstrate that *TWF2* promotes RCC cell invasion, migration, metastasis, and sunitinib resistance by inhibiting the Hippo signaling. Mechanistically, *TWF2* interacts with Yes‐associated protein (YAP) via the binding residues: *TWF2* M99 and YAP M225. By competitively displacing large tumor suppressor kinase 1, *TWF2* prevents YAP ubiquitination and degradation, leading to its stabilization and subsequent nuclear translocation. Mutation of the M99 residue abolishes the tumor‐promoting activity of *TWF2*. Furthermore, salvianolic acid E is identified as a small‐molecule inhibitor of the *TWF2*–YAP interaction, and synergistically enhances sunitinib efficacy in RCC cell lines and patient‐derived xenograft models. These findings highlight *TWF2* as a promising therapeutic target for overcoming drug resistance in RCC.

## Graphical abstract

Twinfilin actin‐binding protein (TWF2) is upregulated in sunitinib‐resistant renal cell carcinoma (RCC) cells, where it interacts with YAP and protects YAP from degradation. Stabilized YAP translocates into the nucleus and activates transcription of target genes, promoting RCC progression and drug resistance. The small‐molecule compound Salvianolic acid E disrupts the TWF2‐YAP interaction and enhances the therapeutic efficacy of sunitinib in RCC.

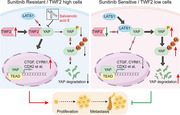

## 1 Introduction

Renal cell carcinoma (RCC) is a highly prevalent malignancy of the urinary system, with epidemiological data indicating a trend among younger patients.^[^ ^1^ ^]^ In 2025, the United States is projected to report 80 980 new cases (52 410 in males and 28 570 in females) and an estimated 14 510 deaths attributable to renal cell carcinoma and renal pelvis cancer.^[^ ^2^ ^]^ Clear cell RCC (ccRCC), the most common histological subtype, accounts for ≈70–90% of RCC cases,^[^ ^3^ ^]^ and is characterized by an insidious onset; only 10% of patients present with the classic symptom triad,^[^ ^4^, ^5^ ^]^ and ≈30% are initially diagnosed with distant metastases. Despite undergoing radical nephrectomy, 20–40% of patients with localized RCC develop recurrence and metastatic progression.^[^ ^6^, ^7^ ^]^ The median survival for patients with metastatic RCC is less than 12 months, and the 5 years survival rate remains between 5% and 12%.^[^ ^6^, ^8^ ^]^ According to European Association of Urology and National Comprehensive Cancer Network guidelines, tyrosine kinase inhibitors (TKIs) are recommended as first‐line therapy for RCC.^[^ ^9^, ^10^ ^]^ However, the clinical efficacy of TKIs remains limited: sunitinib and other targeted agents exhibit intrinsic resistance in 60–70% of cases, and most patients develop acquired resistance within 16 months of treatment initiation.^[^ ^10^, ^11^ ^]^ These resistance mechanisms significantly curtail long‐term clinical benefit. The molecular drivers of RCC progression and metastasis are not yet fully delineated, highlighting the need to investigate resistance pathways and identify novel biomarkers to support the development of more effective individualized therapeutic strategies.

Twinfilin actin‐binding protein (*TWF2*), located on human chromosome 3q21.1, encodes an actin‐binding protein that regulates cytoskeletal dynamics and cellular behavior.^[^ ^12^, ^13^ ^]^ As a key modulator of cell structure and motility,^[^ ^14^ ^]^ *TWF2* plays essential roles in biological processes such as cell migration, mitotic regulation, and signal integration.^[^ ^15^, ^16^, ^17^ ^]^ Recent studies have implicated *TWF2* in multiple malignancies, including breast and non‐small‐cell lung carcinomas.^[^ ^18^ ^]^ *TWF2* overexpression promotes metastatic progression through Rho‐GTPase‐mediated cytoskeletal remodeling and enhanced invasiveness.^[^ ^14^, ^19^, ^20^ ^]^ Despite these insights, the role of *TWF2* in RCC remains poorly defined. Our previous multicohort study (including Chinese, American, and the Cancer Genome Atlas (TCGA) datasets) identified *TWF2* as an independent prognostic biomarker in RCC, with elevated expression significantly correlated with reduced survival overall.^[^ ^21^ ^]^ Therefore, elucidating the tissue‐specific molecular mechanisms and therapeutic potential of *TWF2* in renal malignancies is of considerable importance.

The Hippo signaling pathway, initially discovered in *Drosophila*, is a highly conserved pathway that plays a central role in regulating cell proliferation, apoptosis, and tissue homeostasis.^[^ ^22^, ^23^, ^24^ ^]^ In mammals, this pathway comprises a kinase cascades involving MST1/2 and large tumor suppressor kinase 1/2 (LATS1/2), which regulate the activity of the transcriptional coactivators including Yes‐associated protein (YAP) and TAZ.^[^ ^22^, ^24^, ^25^, ^26^ ^]^ These effectors govern gene expression and cell behavior, influencing both physiological and pathological processes.^[^ ^27^, ^28^ ^]^ Dysregulation of the Hippo pathway is frequently observed in various cancers, promoting tumor development and progression.^[^ ^29^, ^30^, ^31^, ^32^ ^]^ In addition to its roles in proliferation and survival, the Hippo‐YAP/TAZ pathway also contributes to therapeutic resistance, including reduced sensitivity to radiation therapy.^[^ ^33^, ^34^ ^]^ Despite advances in understanding its function in cancer, the mechanisms underlying its interaction with other signaling pathways and biological processes remain incompletely defined, warranting further investigation for the development of targeted therapeutic strategies.

In this study, integrative analyses revealed that *TWF2* is significantly upregulated in RCC tissues, particularly in sunitinib‐resistant cases, and strongly associated with poor prognosis in patients with RCC. In vitro and in vivo experiments demonstrated that *TWF2* promotes RCC progression and sunitinib resistance by interacting with YAP at the specific residues *TWF2* M99 and YAP M225, thereby inhibiting Hippo signaling and stabilizing YAP by preventing ubiquitination and degradation. Salvianolic acid E (Sal E) was identified as a small‐molecule inhibitor of the *TWF2*–YAP interaction, enhancing the efficacy of sunitinib in RCC patient‐derived xenograft (PDX) models. These findings provide novel insights into the molecular basis of RCC progression and drug resistance and suggest *TWF2* as a promising therapeutic target to improve clinical outcomes in RCC.

## 2 Results

### 2.1 Identification of *TWF2* as a Key Regulator for RCC Drug Resistance and Tumor Progression

**Figure 1:**
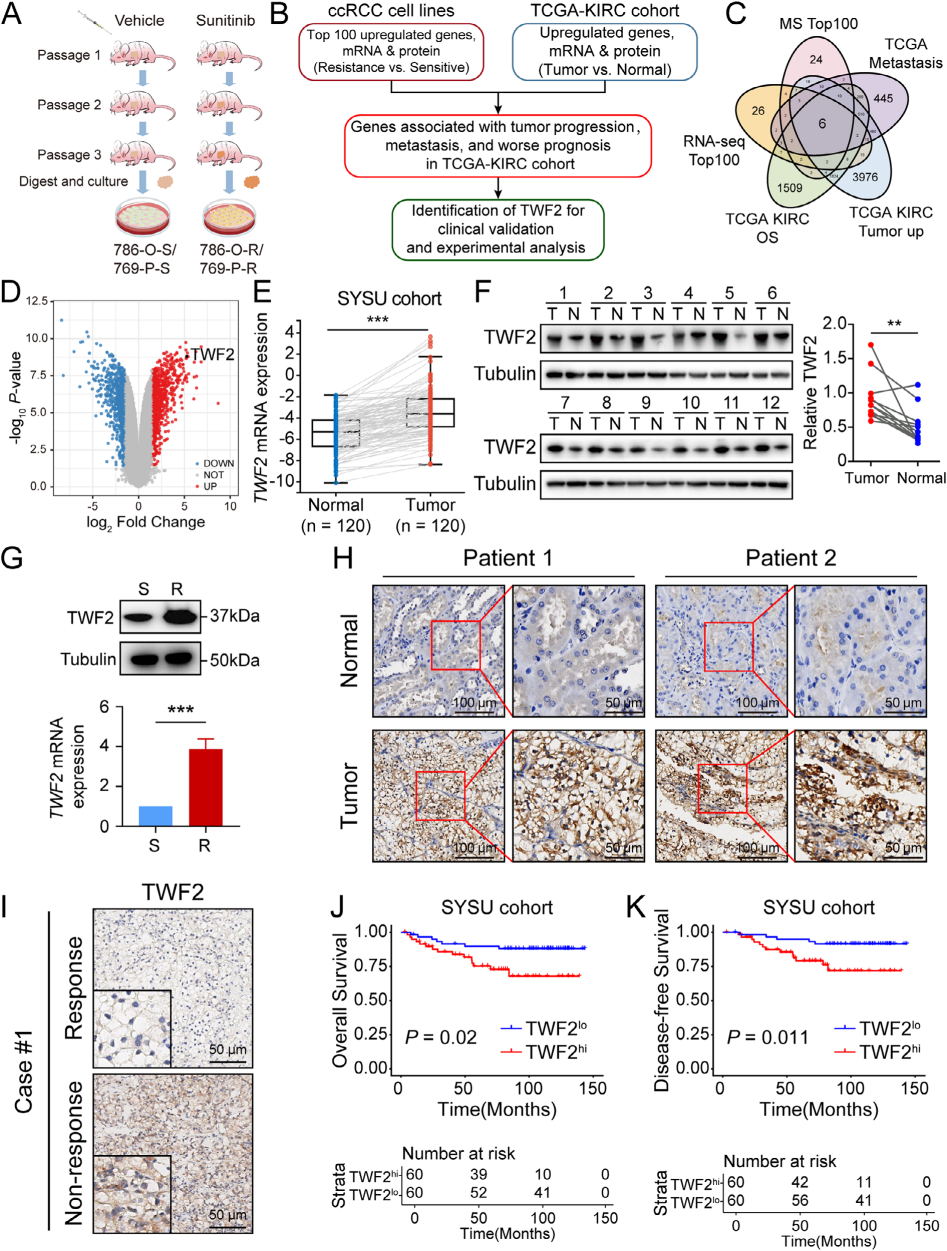
*TWF2* is identified as a master regulator for RCC drug resistance and tumor progression. A) Schematic diagram illustrating the establishment of sunitinib‐resistant (786‐O‐R, 769‐P‐R) and sunitinib‐sensitive (786‐O‐S, 769‐P‐S) RCC cell lines. B) Flow chart of screening key regulators involved in RCC drug resistance and tumor progression. C) Venn diagram displaying the intersection of five datasets: MS Top100, RNA‐seq Top100, TCGA–KIRC OS (genes upregulated in tumors vs normal tissues), TCGA–KIRC Metastasis (genes upregulated in metastatic vs primary tumors), and TCGA–KIRC Prognosis (genes associated with poor prognosis). This analysis identified six overlapping genes: *CTHRC1*, *TWF2*, *COL6A3*, *SLC38A5*, *IFI44*, and *OASL*. D) Volcano plot of differentially expressed genes in sunitinib‐resistant versus sunitinib‐sensitive 786‐O cells based on transcriptomic analysis. Genes upregulated in resistant cells are shown in red, and downregulated genes in blue. E) Relative *TWF2* mRNA expression in paired tumor and adjacent normal tissues from the SYSU ccRCC cohort. F) Representative western blot (left) and the corresponding statistical analysis (right) of *TWF2* protein expression levels in twelve paired ccRCC tumors (T) and adjacent normal tissues (N). G) Representative western blot (top) and mRNA expression analysis (bottom) of *TWF2* in sunitinib‐sensitive (S) and ‐resistant (R) 786‐O cell. H) Representative immunohistochemical (IHC) staining showing *TWF2* expression in ccRCC tumors and adjacent normal tissues. I) Representative IHC images of *TWF2* expression in ccRCC tissues from patients classified as responders or nonresponders to sunitinib treatment. J) Overall survival (OS) in ccRCC patients with low (*n* = 60) or high (*n* = 60) *TWF2* expression. K) Disease‐free survival (DFS) in ccRCC patients with low (*n* = 60) or high (*n* = 60) *TWF2*. Data are presented as means ± SD and are analyzed by Student's *t*‐test (E–G) or log‐rank test (J, K). **p < 0.01; ***p < 0.001.

Sunitinib‐resistant and sensitive control 786‐O and 769‐P cells were established through cycles of sunitinib treatment combined with serial in vivo passaging (**Figure** **1**A). Compared with sensitive controls, the resistant 786‐O and 769‐P cells exhibited increased half‐maximal inhibitory concentration (IC50) values in response to sunitinib (Figure S1A,B, Supporting Information). RNA sequencing and mass spectrometry analysis were subsequently performed on sunitinib‐resistant and ‐sensitive 786‐O cells. An integrative analysis combining sequencing data from RCC cell lines and TCGA–Kidney Renal Clear Cell Carcinoma (KIRC) dataset was conducted to identify key regulatory factors implicated in drug resistance and tumor progression. Genes upregulated at both mRNA and protein levels in resistant 786‐O cells and KIRC tumor tissues were assessed for associations with tumor progression, metastasis, and prognosis. Six genes, Collagen Triple Helix Repeat Containing 1 (*CTHRC1*), *TWF2*, Collagen Type VI Alpha 3 (*COL6A3*), Solute Carrier Family 38 Member 5 (*SLC38A5*), Interferon Induced Protein 44 (*IFI44*), and 2′‐5′ Olive Acid Synthetase Like (*OASL*), were identified as potential master regulators of RCC drug resistance and tumor development (Figure 1B–D; Figure S2A, Supporting Information). The identification of *TWF2* through this integrative analysis highlights its potential role as a master regulator in RCC resistance and progression. A previous study identified *TWF2* as a prognostic biomarker in ccRCC via genome‐wide CpG methylation profiling.^[^ ^21^ ^]^ However, its functional role and mechanistic contributions to drug resistance and tumor progression remain to be elucidated. Analysis of TCGA–KIRC dataset revealed significantly upregulated *TWF2* expression in tumor tissues relative to normal controls (Figure S2B,C, Supporting Information). In the same cohort, *TWF2* expression was elevated with pathological stage (Figure S2D, Supporting Information) and metastatic status (Figure S2E,F, Supporting Information). Validation using the SYSU ccRCC cohort confirmed elevated *TWF2* expression at both mRNA and protein levels in tumor tissues compared to adjacent normal tissues, as demonstrated using real‐time quantitative polymerase chain reaction (RT‐qPCR) and western blotting (Figure 1E,F). RCC cell lines, particularly the sunitinib‐resistant lines, also exhibited increased *TWF2* expression relative to normal HK2 cells (Figure 1G; Figure S2G–I, Supporting Information). Immunohistochemical analysis of paired ccRCC samples from the SYSU cohort further supported the significant upregulation of *TWF2* in tumor tissues, especially among patients with sunitinib resistance (Figure 1H,I; Figure S2J, Supporting Information). In TCGA–KIRC cohort, higher *TWF2* expression was associated with reduced disease‐free survival (DFS) (Figure S2K, Supporting Information). Similarly, the independent ccRCC cohort demonstrated that elevated *TWF2* levels were associated with shorter overall survival (OS) and DFS (Figure 1J,K). These findings establish *TWF2* as a potential predictor of drug resistance, tumor aggressiveness, and unfavorable prognosis in ccRCC.

### 2.2 *TWF2* Promotes Tumor Progression and Sunitinib Resistance in RCC

**Figure 2:**
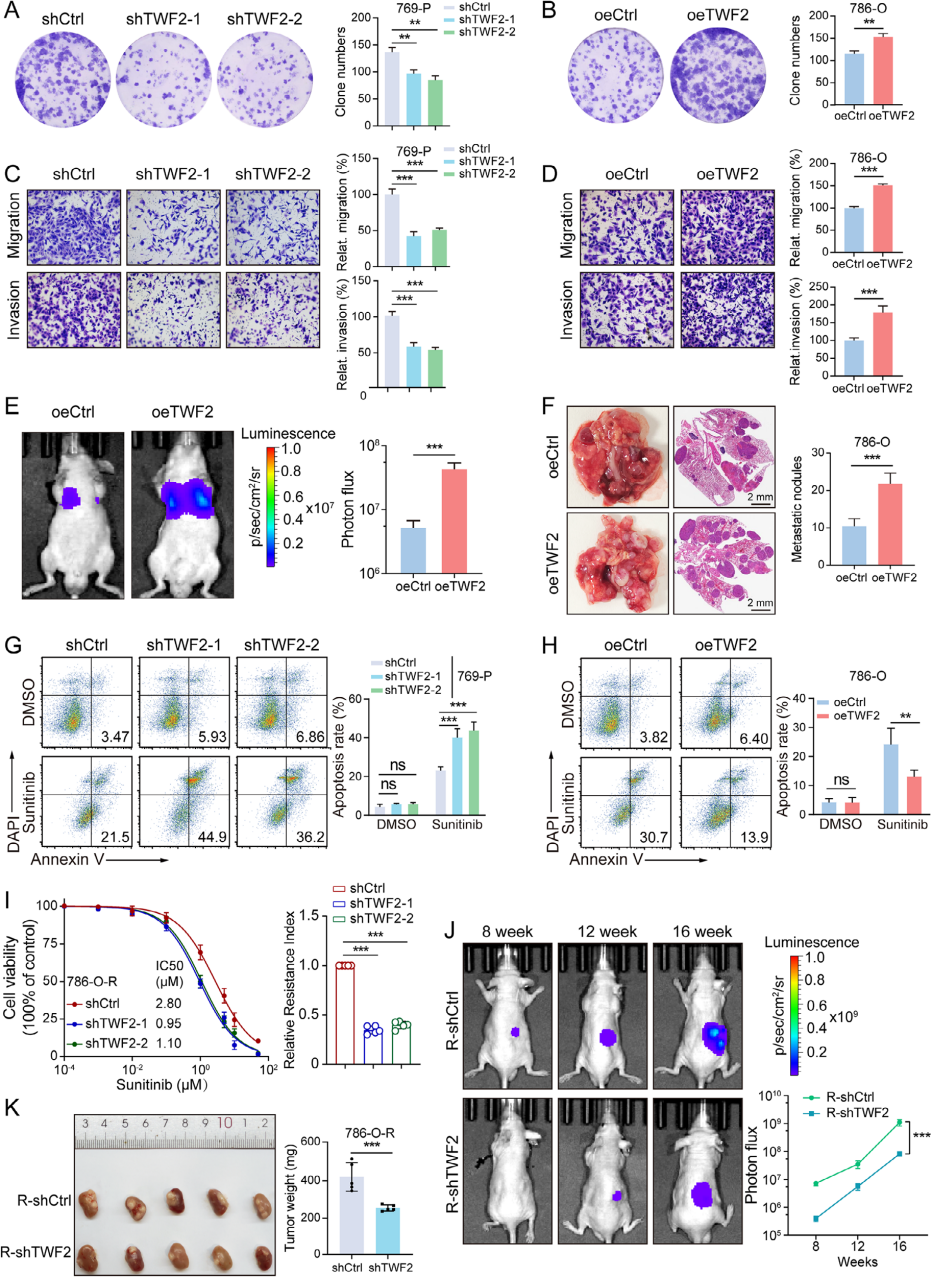
*TWF2* promotes RCC progression and contributes to sunitinib resistance. A,B) Colony formation assay of *TWF2*‐knockdown 769‐P cells (A) and *TWF2*‐overexpressing 786‐O cells (B). C,D) Transwell assays evaluating migration and invasion of *TWF2*‐knockdown 769‐P cells (C) and *TWF2*‐overexpressing 786‐O cells (D). E) Representative bioluminescence images (left) and the corresponding statistical analysis (right) of lung metastases in mice injected with *TWF2*‐overexpressing 786‐O cells. F) Representative gross of lung images and hematoxylin–eosin (H&E) staining of metastatic lesions (left), with statistical analysis (right), from the metastasis model shown in (E). G,H) Flow‐cytometry‐based apoptosis analysis of *TWF2*‐knockdown (G) and *TWF2*‐overexpressing (H) cells and their respective controls following treatment with sunitinib (2 µm) or DMSO for 60 h. I) Relative cell viability (left) and resistance index (right) in *TWF2*‐knockdown and control 786‐O‐R cells treated with sunitinib, based on Cell Counting Kit‐8 (CCK‐8) assays. J) Representative bioluminescence images (left) of orthotopic tumors formed by *TWF2*‐knockdown or control 786‐O‐R cells treated with sunitinib, with the corresponding statistical analysis (right). K) Gross images of orthotopic renal tumors (left) and the corresponding tumor weights (right) from (J). 786‐O‐R: sunitinib‐resistant 786‐O cells. Data are presented as means ± SD and are analyzed by Student's *t*‐test (A–K) or one‐way ANOVA (J). ns, no significance; **p < 0.01; ***p < 0.001.

The biological functions of *TWF2* in RCC were investigated by generating *TWF2*‐knockdown 769‐P cells and *TWF2*‐overexpressing 786‐O cells based on baseline *TWF2* expression in RCC cell lines (Figure S3A, Supporting Information). Cell Counting Kit‐8 (CCK‐8) viability and colony formation assays indicated slight effects of *TWF2* modulation on proliferation and clonogenicity in both cell models (**Figure** **2**A,B; Figure S3B,C, Supporting Information). Wound healing and Transwell assays revealed markedly reduced migratory and invasive capacities in *TWF2*‐knockdown cells, whereas *TWF2*‐overexpression enhanced these properties (Figure 2C,D; Figure S3D,E, Supporting Information). In vivo evaluation using a pulmonary metastasis model established via tail vein injection of *TWF2*‐overexpressing 786‐O cells showed significantly increased pulmonary metastasis and formation of metastatic nodules, confirmed using fluorescence imaging and histological assessment (Figure 2E,F). These findings suggest that *TWF2* enhances RCC progression, particularly cell migration and metastatic dissemination.

The potential role of *TWF2* in mediating sunitinib resistance was further examined. Apoptosis assays showed increased apoptotic rates in *TWF2*‐deficient 769‐P cells and decreased apoptosis in *TWF2*‐overexpressing 786‐O cells following sunitinib treatment (Figure 2G,H). In sunitinib‐resistant 786‐O and 769‐P cells, *TWF2* knockdown suppressed drug resistance, as indicated by reduced viability posttreatment (Figure 2I; Figure S3F,G, Supporting Information). In vivo, an orthotopic RCC tumor model was established using luciferase‐labeled *TWF2*‐knockdown and control 786‐O‐R cells in BALB/c nude mice. Tumor size was monitored weekly using in vivo imaging systems. Sunitinib administration led to significantly reduced tumor growth and volume in the *TWF2*‐deficient group, as monitored weekly via in vivo imaging (Figure 2J,K). These data collectively demonstrate that *TWF2* contributes to RCC cell migration, invasion, metastatic potential, and resistance to sunitinib, supporting its potential as a therapeutic target in drug‐resistant RCC.

### 2.3 *TWF2* Impedes Hippo Signaling by Augmenting the Nuclear Localization of YAP

**Figure 3:**
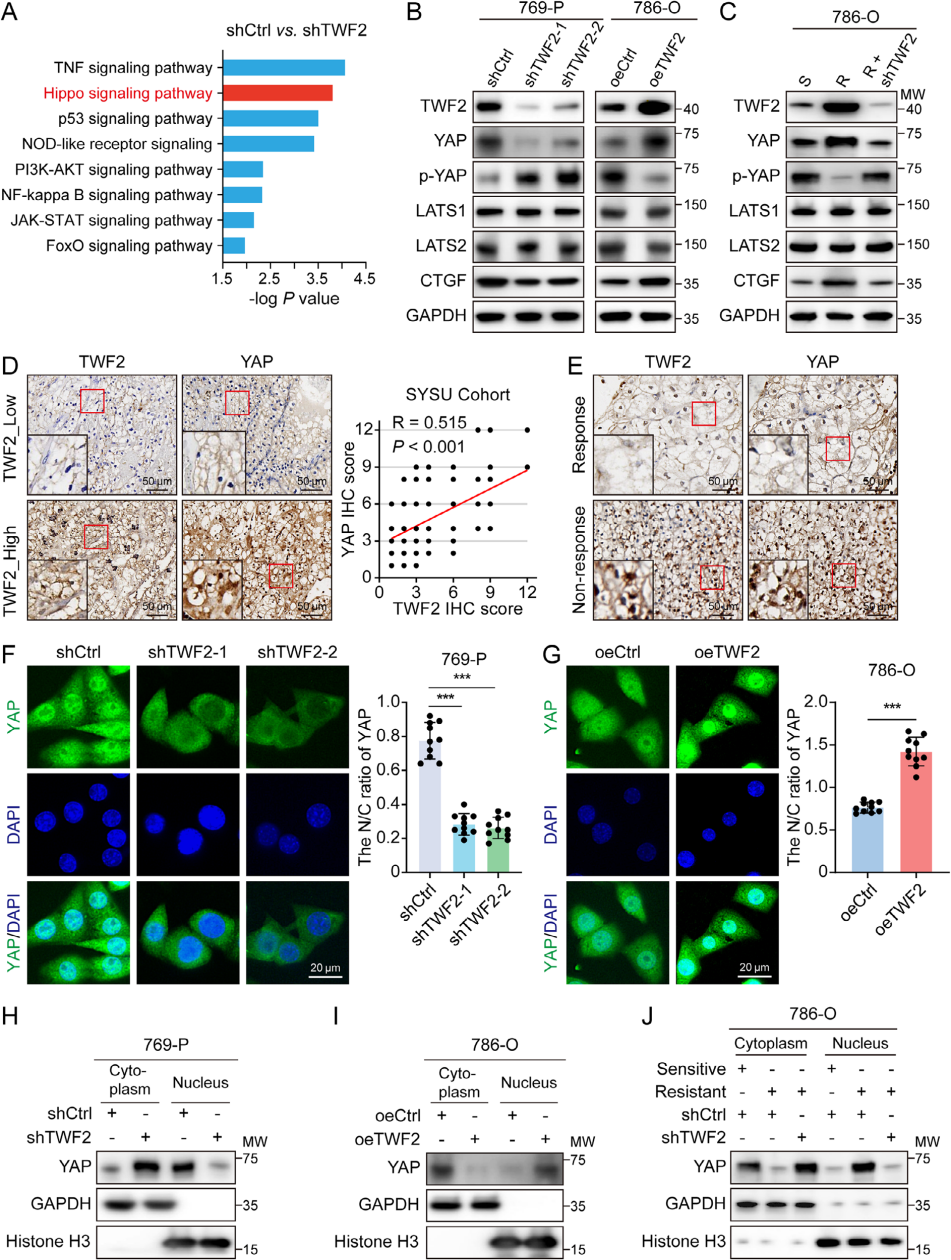
*TWF2* inhibits Hippo signaling through enhancing dephosphorylation and nuclear translocation of YAP. A) KEGG pathway enrichment analysis of differentially expressed genes following *TWF2* knockdown in 769‐P cells, indicating altered Hippo signaling. B) Western blot analysis of Hippo signaling pathway components in *TWF2*‐knockdown 769‐P cells and *TWF2*‐overexpressing 786‐O cells. C) Western blot analysis of Hippo‐pathway‐associated proteins in sunitinib‐sensitive (S), sunitinib‐resistant (R), and *TWF2*‐knockdown sunitinib‐resistant (R + sh*TWF2*) 786‐O cells. D) Representative IHC images (left) and correlation analysis (right) between *TWF2* and YAP based on their expression in human ccRCC tissues. E) Representative immunohistochemical images of *TWF2* and YAP in human ccRCC tissues from sunitinib‐responsive and nonresponsive patients. F) Representative immunofluorescence images (left) and quantification (right) showing YAP subcellular localization following *TWF2* knockdown. G) Representative immunofluorescence images (left) and quantification (right) showing YAP subcellular localization following *TWF2* overexpression. H,I) Western blot analysis of nuclear and cytoplasmic YAP distribution in *TWF2*‐knockdown 769‐P cells (H) and *TWF2*‐overexpressing 786‐O cells (I). J) Western blot analysis of nuclear and cytoplasmic YAP levels in the indicated cell lines. Data are presented as means ± SD and are analyzed by Student's *t*‐test (F, G) or Pearson correlation test (D). ***p < 0.001.

The mechanism underlying *TWF2*‐mediated RCC progression and drug resistance was examined using RNA sequencing of total RNA from *TWF2*‐knockdown and control 769‐P cells. Kyoto Encyclopedia of Genes and Genomes (KEGG) pathway analysis identified significant dysregulation of the Hippo signaling pathway following *TWF2* depletion (**Figure** **3**A; Figure S4A, Supporting Information). Gene set enrichment analysis (GSEA) revealed negative association between *TWF2* expression and Hippo signaling (Figure S4B, Supporting Information). YAP phosphorylation, particular at Ser127, serves as a key indicator of Hippo pathway activity. When activated, LATS1/2 kinases phosphorylate YAP at Ser127, leading to cytoplasmic retention and suppression of transcriptional activity. By contrast, unphosphorylated YAP translocates into the nucleus and interacts with TEA domain (TEAD) transcription factors to drive oncogenic gene expression. Phosphorylation at Ser381 enables CK1δ/ε‐mediated priming, followed by recognition by the E3 ubiquitin ligase SCFβ‐TRCP, resulting in YAP ubiquitination and proteasomal degradation.^[^ ^35^ ^]^ Additional phosphorylation site also contributes to Hippo pathway regulation.^[^ ^34^, ^36^, ^37^, ^38^, ^39^, ^40^, ^41^, ^42^ ^]^ *TWF2* overexpression elevated total YAP protein level, and reduced phosphorylation at Ser127 and Ser381, with minimal impact on phosphorylation at Ser61 and Ser109 (Figure 3B; Figure S4C, Supporting Information). Although LATS1/2 expression remained unchanged, connective tissue growth factor (*CTGF*), a canonical YAP target gene, was significantly upregulated (Figure 3B). The mRNA levels of *CTGF*, *CYR61*, and *CDX2* were downregulated upon *TWF2* knockdown and upregulated with *TWF2* overexpression (Figure S4D,E, Supporting Information). Sunitinib‐resistant 786‐O and 769‐P cells exhibited elevated expression of *TWF2* and YAP, accompanied by reduced levels of phosphorylated YAP (p‐YAP) and increased *CTGF* expression, relative to sunitinib‐sensitive cells. *TWF2* knockdown in resistant cells restored p‐YAP expression and reduced total YAP and *CTGF* levels (Figure 3C; Figure S4F, Supporting Information), implying that *TWF2* suppresses YAP phosphorylation and enhances its transcriptional activity in the resistant phenotype.

In ccRCC tissues, immunohistochemical analysis demonstrated a positive correlation between *TWF2* and YAP expression (Figure 3D). Both proteins were weakly expressed in sunitinib‐responsive samples and strongly expressed in resistant tissues (Figure 3E).

Given the role of phosphorylation in regulating YAP subcellular localization, immunofluorescence imaging was performed. Nuclear YAP was diminished in *TWF2*‐knockdown 769‐P cells (Figure 3F) and increased in *TWF2*‐overexpressing 786‐O cells (Figure 3G). Subcellular fractions confirmed reduced nuclear YAP in *TWF2*‐deficient cells and enhanced nuclear YAP in *TWF2*‐overexpressing cells compared with controls (Figure 3H,I). In resistant cells, *TWF2* depletion reversed the enhanced nuclear YAP localization (Figure 3J), suggesting a regulatory role for *TWF2* in YAP nuclear translocation. Overall, *TWF2* inactivated Hippo signaling by impairing YAP phosphorylation and promoting its nuclear localization in RCC.

### 2.4 *TWF2* Interacts with YAP via the WW Domain

**Figure 4:**
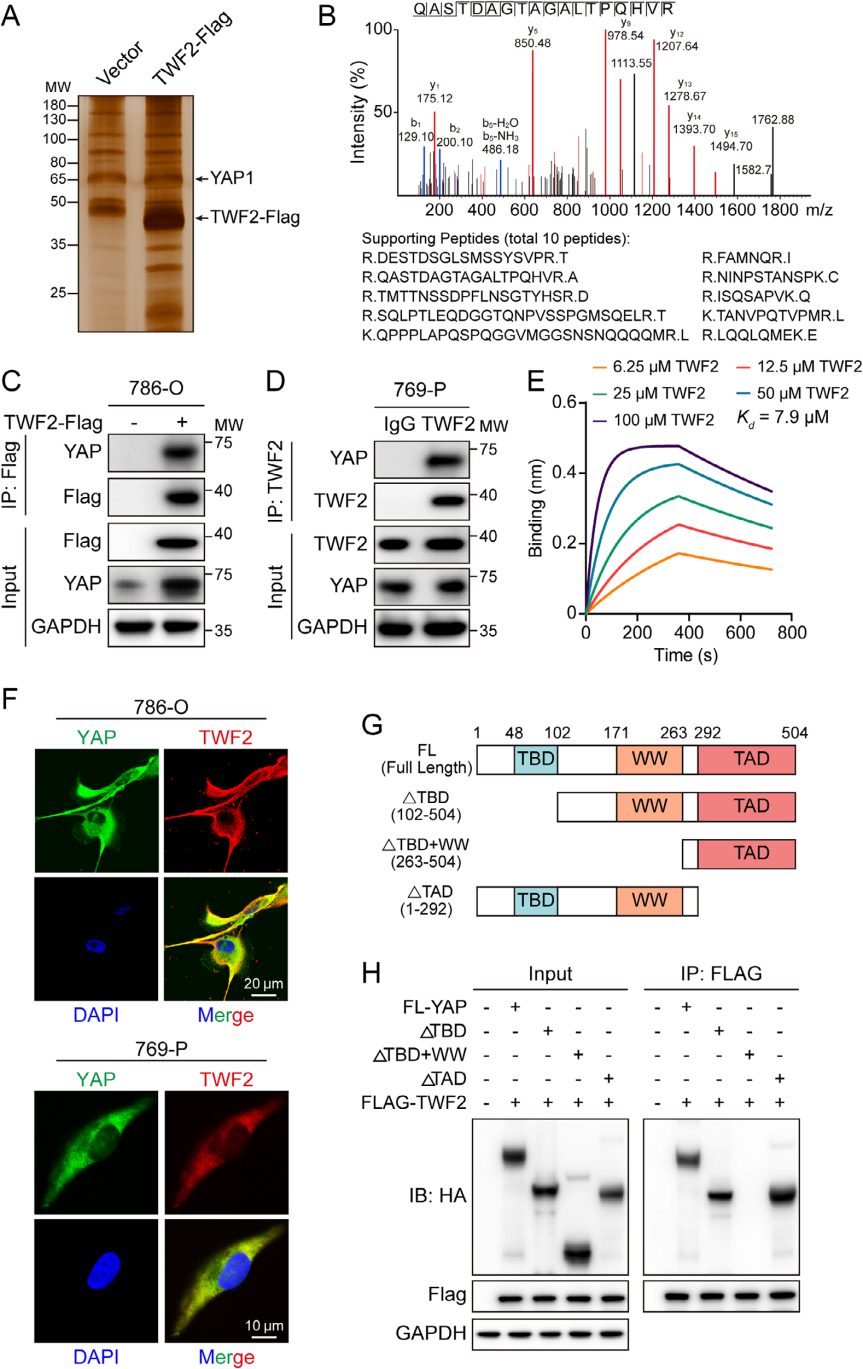
Identification of YAP as a binding partner of *TWF2* in RCC cells. A) Representative silver‐stained gel showing proteins specifically immunoprecipitated by the *TWF2* protein. B) LC–MS/MS analysis identified YAP as a *TWF2*‐interacting protein in Flag‐*TWF2* immunoprecipitated samples. C) Co‐IP of overexpressed Flag‐tagged *TWF2* and endogenous YAP in 786‐O cells. D) Co‐IP of endogenous *TWF2* and YAP in 769‐P cells. E) BLI assay measuring the binding affinity between purified *TWF2* and YAP. F) Representative immunofluorescent images showing colocalization of endogenous *TWF2* and YAP in 786‐O (top) and 769‐P (bottom) cells. G) Schematic diagrams of wild‐type YAP (full‐length, 1–504) and its truncation mutants. H) Co‐IP analysis demonstrating interactions between *TWF2* constructs and YAP. 786‐O cells transfected with Flag‐*TWF2* and the indicated YAP constructs with HA‐tag were subjected to immunoprecipitation using an anti‐Flag antibody, followed by immunoblotting with anti‐HA and anti‐Flag antibodies.

The mechanism by which *TWF2* mediates YAP phosphorylation and nuclear translocation was investigated under the premise that *TWF2*‐binding proteins may contribute to this process. Flag‐tagged *TWF2* (*TWF2*‐Flag) was overexpressed in 786‐O cells, and binding proteins were isolated using co‐immunoprecipitation (Co‐IP) and visualized using silver staining. Sodium dodecyl sulfate–polyacrylamide gel electrophoresis (SDS‐PAGE) revealed a distinct band at ≈70 kDa (**Figure** **4**A), which was excised and subjected using liquid chromatography–mass spectrometry (LC–MS). YAP was identified as a putative binding protein of *TWF2* (Figure 4B). The interaction between *TWF2* and YAP was confirmed through Co‐IP assays, showing that they bind directly both when overexpressed in 786‐O cells (Figure 4C) and at endogenous levels in 769‐P cells (Figure 4D). Biolayer interferometry (BLI) further confirmed direct in vitro binding between YAP proteins and recombinant *TWF2* (Figure 4E). Immunofluorescence staining demonstrated colocalization of *TWF2* and YAP in the cytoplasm of 786‐O and 769‐P cells (Figure 4F).

YAP is a transcriptional coactivator comprising a TEAD‐binding domain (TBD), WW domain, and transcriptional activation domain (TAD). A series of N‐terminal HA‐tagged YAP deletion mutants were generated for delineating the binding region with *TWF2* (Figure 4G). Coexpression of full‐length or truncated mutants of YAP with *TWF2*‐Flag in 786‐O cells, followed by Co‐IP, indicating that the WW domain mediates the *TWF2*–YAP interaction (Figure 4H). Collectively, these findings reveal that *TWF2* directly binds to YAP via the WW domain in RCC cells.

### 2.5 *TWF2* Stabilizes YAP by Interfering with LATS1‐Mediated YAP Phosphorylation and Subsequent Ubiquitin‐Proteasomal Degradation

**Figure 5:**
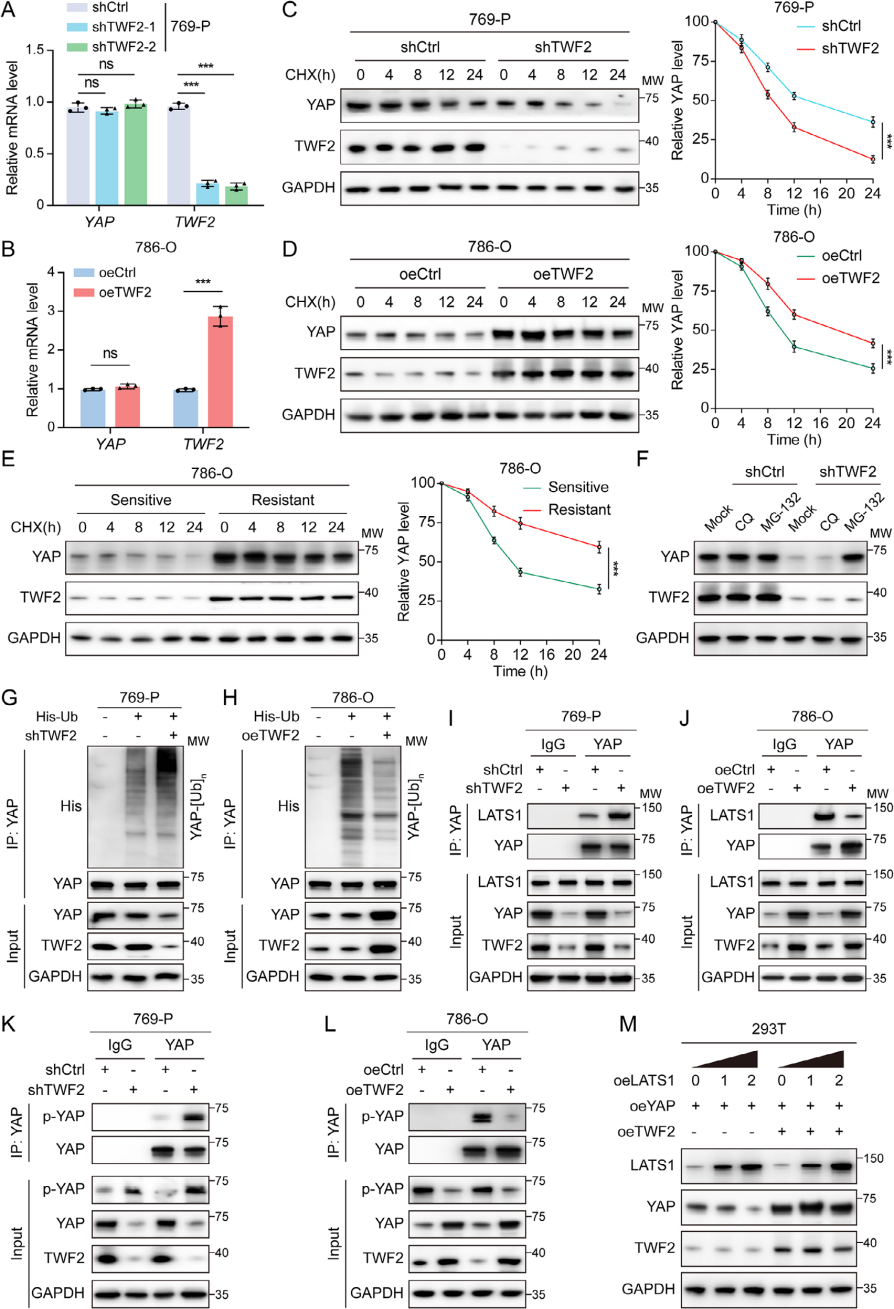
*TWF2* binding to YAP enhances YAP stability by competing with LATS1. A,B) Quantitative RT‐PCR analysis of YAP mRNA levels in *TWF2* knockdown 769‐P cells (A) and *TWF2*‐overexpressing 786‐O cells (B). C–E) Western blot analysis of YAP protein stability using CHX chase assays in *TWF2* knockdown 769‐P cells (C), *TWF2*‐overexpressing 786‐O cells (D), and sunitinib‐sensitive versus ‐resistant 786‐O cells (E). Cells were treated with 20 µg mL^−1^ CHX for the indicated time points. Statistical analyses are shown in the right panels. F) Western blot showing YAP protein levels in *TWF2* knockdown and control 769‐P cells following treatment with chloroquine (CQ, 10 µm) or MG132 (10 µm) for 12 h. G,H) Co‐IP assays showing increased YAP ubiquitination in *TWF2*‐deficient 769‐P cells (G) and decreased YAP ubiquitination in *TWF2*‐overexpressing 786‐O cells (H). I,J) Co‐IP assays with anti‐YAP antibody showing the precipitated LATS1 levels in *TWF2*‐knockdown 769‐P cells (I) and *TWF2*‐overexpressing 786‐O cells (J). K,L) Co‐IP assays with anti‐YAP antibody showing p‐YAP levels in *TWF2* knockdown 769‐P cells (K) and *TWF2*‐overexpressing 786‐O cells (L). M) Western blot showing the effect of increasing LATS1 and *TWF2* expression on exogenous YAP levels in 293T cells transfected with the indicated plasmids. Data are presented as means ± SD and are analyzed by Student's *t*‐test (A, B) or one‐way ANOVA (C–E). ns, no significance; ***p < 0.001.

The biological effects of the *TWF2*–YAP interaction were investigated to delineate its regulatory effect on YAP stability. Overexpression of *TWF2* resulted in increased YAP protein level (Figure 3B), while mRNA levels remained unchanged following either *TWF2* knockdown or overexpression (**Figure** **5**A,B), suggesting a posttranscriptional mode of regulation. The observed reduction in YAP protein following *TWF2* knockdown implied a potential role in modulating YAP degradation. YAP stability was evaluated following cycloheximide (CHX) treatment to block protein synthesis. YAP underwent rapid degradation in *TWF2*‐knockdown 769‐P cells (Figure 5C), whereas its half‐life was significantly extended in *TWF2*‐overexpressing cells (Figure 5D), indicating that *TWF2* enhances YAP protein stability. In sunitinib‐resistant 786‐O and 769‐P cells, CHX treatment resulted in delayed YAP degradation relative to their sensitive counterparts (Figure 5E; Figure S5A, Supporting Information), resembling the stabilizing effect observed in *TWF2*‐overexpressing cells. The proteasome and autophagy‐lysosome pathways represent two primary degradation pathways in eukaryotic cells.^[^ ^43^ ^]^ *TWF2*‐knockdown and control cells were treated with the lysosomal inhibitor chloroquine (CQ) or the proteasome inhibitor MG132. MG132 reversed *TWF2* knockdown‐induced YAP degradation, whereas CQ had no effect (Figure 5F), suggesting that *TWF2*‐related YAP stabilization occurs via a proteasome‐dependent mechanism. Ubiquitination assays demonstrated enhanced polyubiquitination of YAP following *TWF2* knockdown, whereas polyubiquitination was reduced under *TWF2* overexpression (Figure 5G,H), indicating that *TWF2* inhibits ubiquitin‐mediated YAP degradation.

YAP is typically phosphorylated by LATS1, resulting in cytoplasmic retention and proteasomal degradation.^[^ ^44^ ^]^ Given these observations, *TWF2* may inhibit YAP ubiquitination by blocking its phosphorylation through competitive interference with LATS1. Co‐IP assays revealed enhanced interaction between LATS1 and YAP in *TWF2*‐knockdown RCC cells (Figure 5I), while this interaction was attenuated in *TWF2*‐overexpressing cells (Figure 5J), suggesting that *TWF2* competes with LATS1 for YAP binding. Consistently, levels of p‐YAP were increased in *TWF2*‐knockdown cells (Figure 5K) and decreased in *TWF2*‐overexpressing RCC cells (Figure 5L), further supporting the role of *TWF2* in inhibiting LATS1‐mediated YAP phosphorylation. Moreover, coexpression experiments in 293T cells demonstrated that LATS1 reduced YAP protein levels in a dose‐dependent manner, while this inhibitory effect was antagonized by *TWF2* overexpression (Figure 5M). Collectively, these data indicate that *TWF2* competes with LATS1 for YAP interaction, thereby preventing YAP phosphorylation and subsequent ubiquitin‐mediated degradation.

The functional importance of YAP in mediating *TWF2*‐driven tumor progression and drug resistance was further validated. YAP overexpression largely rescued the impaired proliferation, invasion, and migration of 769‐P cells caused by *TWF2* loss (Figure S6A–E, Supporting Information). Similarly, the enhanced sensitivity of *TWF2*‐knockdown 769‐P cells to sunitinib was reversed by YAP overexpression (Figure S6F, Supporting Information). Conversely, the malignancy and resistance to sunitinib that were enhanced by *TWF2* overexpression in 786‐O cells were abolished when YAP was depleted (Figure S6G–L, Supporting Information). These findings collectively elucidate the pivotal role of YAP as a downstream effector of *TWF2* in promoting RCC progression and resistance to sunitinib.

### 2.6 Met99 of *TWF2* Is Required for Binding to YAP in RCC Cells

**Figure 6:**
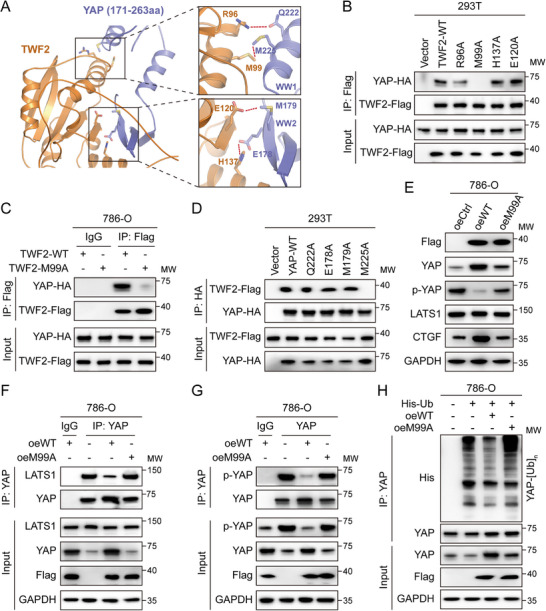
*TWF2* binds to YAP in a Met99‐dependent manner. A) Structure model of the *TWF2*–YAP complex generated using the HDOCK server, indicating predicted binding sites. B) Four amino acids in *TWF2* predicted to mediate binding were individually mutated to alanine. The interaction between mutant *TWF2* and YAP was assessed using Co‐IP in 293T cells using an anti‐Flag antibody. C) Co‐IP assay showing the effect of the *TWF2* M99A mutation on its interaction with YAP in 786‐O cells. D) Four YAP residues predicted as *TWF2*‐binding sites were individually mutated to alanine. Their interaction with *TWF2* was evaluated using Co‐IP in 293T cells using an anti‐HA antibody. E) Western blotting analysis of p‐YAP and Hippo signaling components in 786‐O cells overexpressing either Flag‐tagged wild‐type *TWF2* or the M99A mutant. F) Co‐IP assays with anti‐YAP antibody showing the level of coprecipitated LATS1 in cells overexpressing wild‐type or M99A mutant *TWF2*. G) Co‐IP assays with anti‐YAP antibody detecting the level of coprecipitated p‐YAP in 786‐O cells overexpressing wild‐type or M99A mutant *TWF2*. H) YAP ubiquitination levels in 786‐O cells cotransfected with His‐ubiquitin and either Flag‐tagged wild‐type *TWF2* or the M99A mutant. Cell lysates were subjected to IP with anti‐YAP antibody. oeWT, overexpression of wild‐type *TWF2*; oeM99A, overexpression of *TWF2* M99A mutant.

The disruption of the *TWF2*–YAP interaction was investigated to determine its functional relevance in regulating YAP degradation in RCC cells. Molecular docking was performed using the HDOCK server to predict potential binding interfaces between *TWF2* and YAP, generating a structural interaction model (Figure S7A, Supporting Information). Four pairs of putative interacting residues were identified: *TWF2* Arg96 (R96) with YAP Gln222 (Q222), *TWF2* Met99 (M99) with YAP Met225 (M225), *TWF2* His137 (H137) with YAP Glu178 (E178), *TWF2* Glu120 (E120) with YAP Met179 (M179) (**Figure** **6**A). Each of the four predicted residues of *TWF2* was individually mutated into Alanine (A) and cotransfected with YAP in 293T cells. Co‐IP assays demonstrated that only the M99A mutation disrupted the *TWF2*–YAP interaction (Figure 6B). This finding was corroborated in 786‐O cells (Figure 6C). Correspondingly, YAP mutants were generated and cotransfected with *TWF2* in 293T cells. The M225A mutation in YAP disrupted its binding to *TWF2* (Figure 6D), indicating that the *TWF2*–YAP interaction is specifically mediated through the M99 and M225 residues, respectively.

The functional relevance of the M99 residue was further examined by overexpressing *TWF2* M99A in 786‐O cells. Unlike wild‐type *TWF2*, the M99A mutant failed to reduce p‐YAP levels or elevate YAP and *CTGF* expression (Figure 6E). Co‐IP assays further demonstrated that *TWF2* M99A did not impair the interaction between YAP and LATS1 (Figure 6F), nor did it alter p‐YAP levels (Figure 6G), in contrast to the effects observed with wild‐type *TWF2*. Furthermore, YAP polyubiquitination remained unchanged in the presence of *TWF2* M99A, in contrast to the reduced ubiquitination observed with wild‐type *TWF2* (Figure 6H). Collectively, these findings demonstrate that Met99 of *TWF2* is critical for mediating the interaction with YAP and for protecting YAP from ubiquitin‐mediated degradation.

### 2.7 Met99 of *TWF2* Mediates Its Function in Promoting RCC Progression and Sunitinib Resistance In Vitro and In Vivo

**Figure 7:**
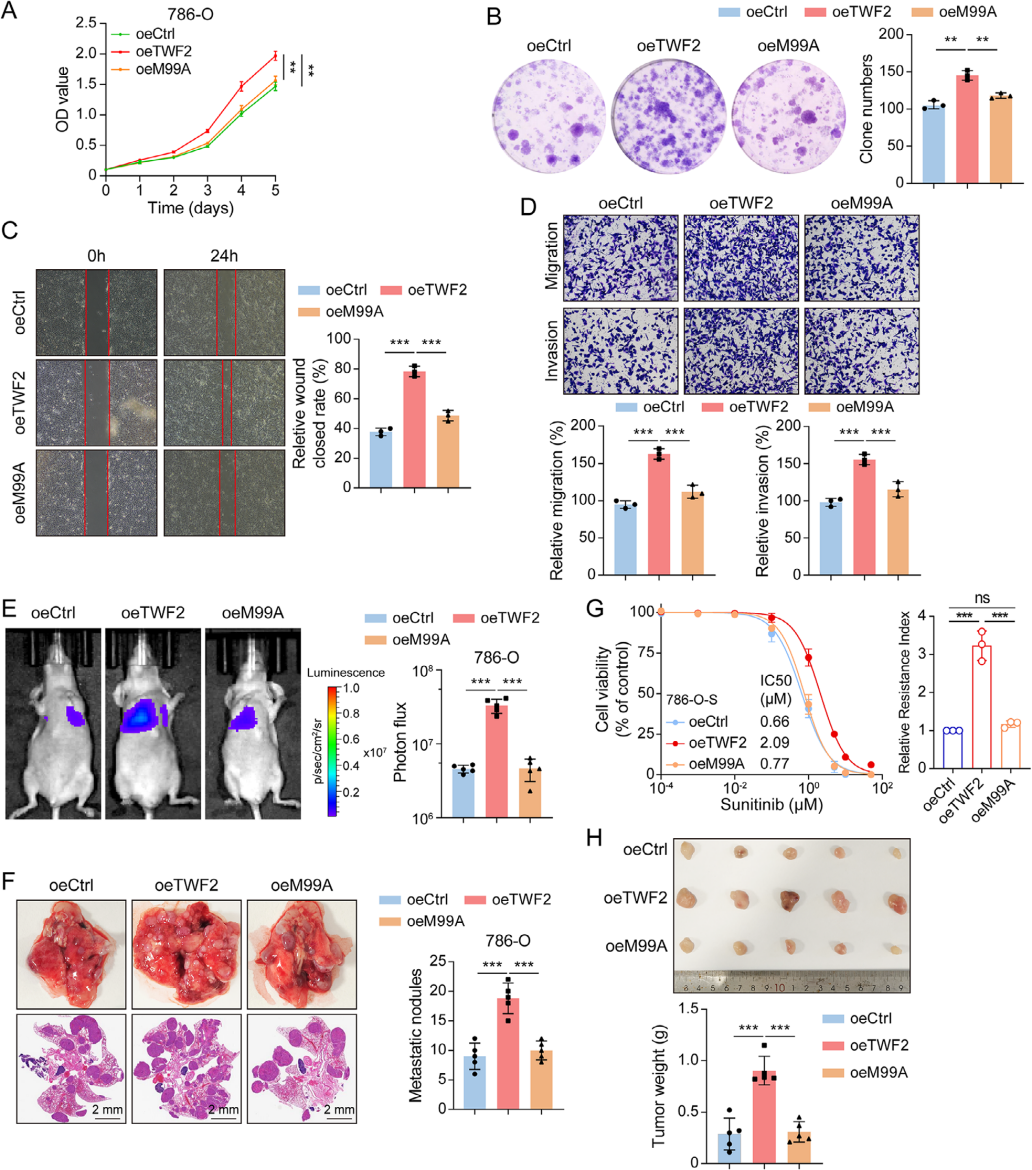
M99A mutation inhibits RCC progression and sunitinib resistance in vitro and in vivo. A) CCK‐8 proliferation assays measuring cell proliferation in 786‐O cells overexpressing wild‐type *TWF2*, the M99A mutant, or control vector. B) Colony formation assay comparing the clonogenic potential of 786‐O cells overexpressing wild‐type *TWF2*, the M99A mutant, or control vector. C) Wound healing assay evaluating the migratory capacity of 786‐O cells overexpressing wild‐type *TWF2*, the M99A mutant, or control vector. D) Transwell assays assessing migration and invasion of 786‐O cells overexpressing wild‐type *TWF2*, the M99A mutant, or control vector. E) Representative bioluminescence images (left) and statistical analysis (right) of lung metastases in a nude mouse model established using the indicated 786‐O cells (*n* = 5 per group). F) Representative images of lungs with metastatic nodules and H&E‐stained sections of metastatic lesions (left), with the corresponding quantification (right) from the metastasis model in (E). G) Relative cell viability (left) and resistance index (right) of 786‐O cells overexpressing control vector, wild‐type *TWF2*, or M99A mutant following sunitinib treatment, measured using CCK‐8 assay. H) Representative images of tumors (top) and tumor weights (bottom) from nude mice bearing sunitinib‐sensitive 786‐O tumors overexpressing control vector, wild‐type *TWF2*, or M99A mutant (*n* = 5 per group) following sunitinib treatment. Data are presented as means ± SD and are analyzed by one‐way ANOVA (A) or Student's *t‐*test (B–H). ns, no significance; **p < 0.01; ***p < 0.001.

The biological functional relevance of the *TWF2*–YAP interaction via the Met99 was assessed by examining the effect of the *TWF2* M99A mutation on RCC cell behavior. CCK‐8 and colony formation assays showed that overexpression of *TWF2* M99A mutant did not increase the proliferation of 786‐O cells, unlike wild‐type *TWF2* (**Figure** **7**A,B). Wound healing and transwell assays further showed that wild‐type *TWF2* increased RCC cell migration and invasion, whereas the M99A mutant lacked this effect (Figure 7C,D). In the pulmonary metastasis model, *TWF2* mutation on M99 significantly impaired the ability of RCC cells to form metastatic lung lesions, indicating that the Met99 residue is required for *TWF2*‐mediated metastatic activity (Figure 7E,F). These findings support the role of Met99 in mediating RCC progression and suggest that it may also be essential for *TWF2*‐driven sunitinib resistance. Drug sensitivity assays in sunitinib‐sensitive 786‐O and 769‐P cells showed that *TWF2* M99A overexpression did not confer resistance, whereas wild‐type *TWF2* markedly reduced drug sensitivity (Figure 7G; Figure S8A,B, Supporting Information). In vivo validation using a xenograft mouse model demonstrated that overexpression of wild‐type *TWF2* resulted in increased tumor burden following sunitinib treatment, whereas the M99A mutant failed to induce resistance (Figure 7H). Collectively, these results establish Met99 as a critical mediator of *TWF2*‐driven tumor progression and therapeutic resistance in RCC.

### 2.8 Small‐Molecule Inhibitor Targeting the *TWF2*–YAP Interaction Enhances the Efficacy of Sunitinib in a ccRCC PDX Model

**Figure 8:**
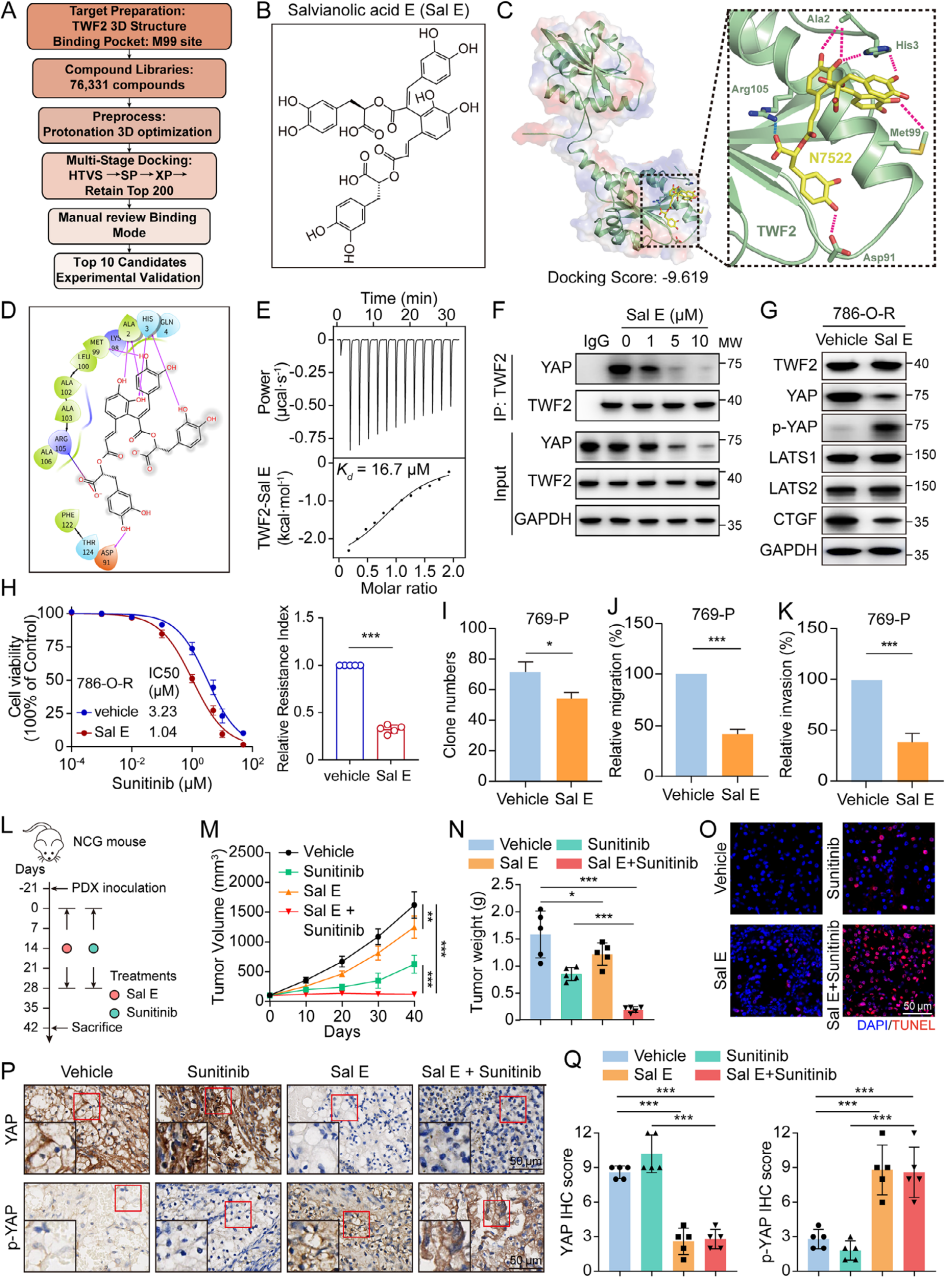
Salvianolic acid E disrupts the *TWF2*–YAP interaction and enhances sunitinib efficacy in RCC PDX models. A) Flowchart of virtual screening for small‐molecule inhibitors targeting *TWF2*–YAP interaction. B) Chemical structure of Sal E. C) 3D structural model of the *TWF2*–Sal E binding interface. The docking score of Sal E for *TWF2* is −9.619. D) 2D interaction map depicting the binding between Sal E and *TWF2*. E) ITC analysis quantifying the binding affinity between *TWF2* and Sal E. The fitted curve yields a dissociation constant (*K*_d_) of 16.7 µm. F) Co‐IP analysis demonstrating disruption of the *TWF2*–YAP interaction in 786‐O‐R cells treated with increasing concentrations of Sal E. G) Western blot analysis of Hippo signaling components in 786‐O‐R cells treated with Sal E. H) Relative cell viability (left) and resistance index (right) of 786‐O‐R cells treated with Sal E or vehicle in combination with sunitinib, as determined using CCK‐8 assay. I–K) Colony formation (I), migration (J), and invasion (K) assays in 769‐P cells treated with Sal E or vehicle. L) Experimental timeline for RCC PDX mouse model treatment with Sal E, sunitinib, or both. Day −21 indicates the day of PDX tumor implantation. M,N) Tumor growth curves (M) and tumor weights (N) in PDX mice treated with vehicle, Sal E, sunitinib, or combination therapy (*n* = 5 per group). O) TUNEL staining showing apoptotic cells in tumors from the indicated treatment groups. P,Q) Representative IHC staining for YAP and p‐YAP (P) and the corresponding quantification of IHC scores (Q) in tumors from treated PDX mice. Sal E, Salvianolic acid E; 786‐O‐R, sunitinib‐resistant 786‐O cells. Data are presented as means ± SD and are analyzed by Student's *t*‐test (H–K, N, Q) or one‐way ANOVA (M). *p < 0.05; **p < 0.01; ***p < 0.001.

Given the critical role of *TWF2* in promoting RCC progression and sunitinib resistance via YAP interaction, the therapeutic potential of targeting *TWF2* was investigated in human RCC models. The structural model of *TWF2* was predicted using AlphaFold, and the M99 residue was selected as the binding site for structure‐based virtual screening of 76 331 compounds from commercial databases. Sal E exhibited the highest docking score and was prioritized for further investigation (**Figure** **8**A,B). 2D and 3D structural analysis indicated that Sal E formed six hydrogen bonds with *TWF2* residues Asp91, Met99, Ala2, and His3, along with one salt bridge at Arg105 (Figure 8C,D). Isothermal titration calorimetry (ITC) confirmed direct binding affinity between Sal E and purified *TWF2*, with a dissociation constant (*K*_d_) of 16.7 µm (Figure 8E). Optimal in vitro concentration was determined by assessing YAP expression across a concentration gradient of Sal E (0–30 µm). Significant downregulation of YAP protein was observed at concentrations ≥10 µm (Figure S9A, Supporting Information), and 10 µm was used in subsequent assays to minimize cytotoxicity. Co‐IP revealed reduced *TWF2*–YAP binding following Sal E treatment in 786‐O‐R cells, indicating disruption of their interaction (Figure 8F). In sunitinib‐resistant 786‐O‐R and 769‐P‐R cells, Sal E increased p‐YAP levels and inhibited Hippo pathway activity (Figure 8G; Figure S9B, Supporting Information). Cell viability assays showed that Sal E reduced resistance in both cell lines (Figure 8H; Figure S9C, Supporting Information). Furthermore, colony formation and transwell assays confirmed that Sal E suppressed clonal expansion, migration, and invasion in 769‐P cells (Figure 8I–K).

Target specificity was validated using ITC, showing loss of Sal E binding upon M99 mutation in *TWF2* (Figure S9D, Supporting Information). In sunitinib‐resistant 786‐O cells with *TWF2* knockdown, Sal E treatment did not alter cell viability, in contrast to dose‐dependent viability reductions in control cells (Figure S9E, Supporting Information). Functional assays corroborated that Sal E suppressed colony formation, migration, and invasion in control cells, whereas no such effect was observed in *TWF2*‐deficient cells (Figure S9F–H, Supporting Information), confirming *TWF2*‐dependent activity.

For the in vivo study, a patient‐derived ccRCC PDX mouse model was used.^[^ ^45^, ^46^, ^47^ ^]^ Mice bearing tumors of similar volumes were treated with escalating doses of Sal E (0, 1, 2.5, 5, 10, 20, and 30 mg kg^−1^). YAP suppression was significant at ≥10 mg kg^−1^ (Figure S9I, Supporting Information), and 10 mg kg^−1^ was selected for subsequent treatments to avoid toxicity. Combination therapy with Sal E and sunitinib was administered, and tumor growth was monitored until the mice were euthanized at six weeks (Figure 8L). The combination yielded the greatest tumor suppression compared to monotherapies (Figure 8M,N). TUNEL staining showed maximal apoptosis in the combination group (Figure 8O), and immunohistochemistry demonstrated reduced YAP and elevated p‐YAP expression in tumors from Sal E‐treated animals (Figure 8P,Q). Collectively, these findings establish Sal E as a selective inhibitor of the *TWF2*–YAP interaction and support its therapeutic potential for enhancing sunitinib efficacy in RCC.

## 3 Discussion

**Figure 9:**
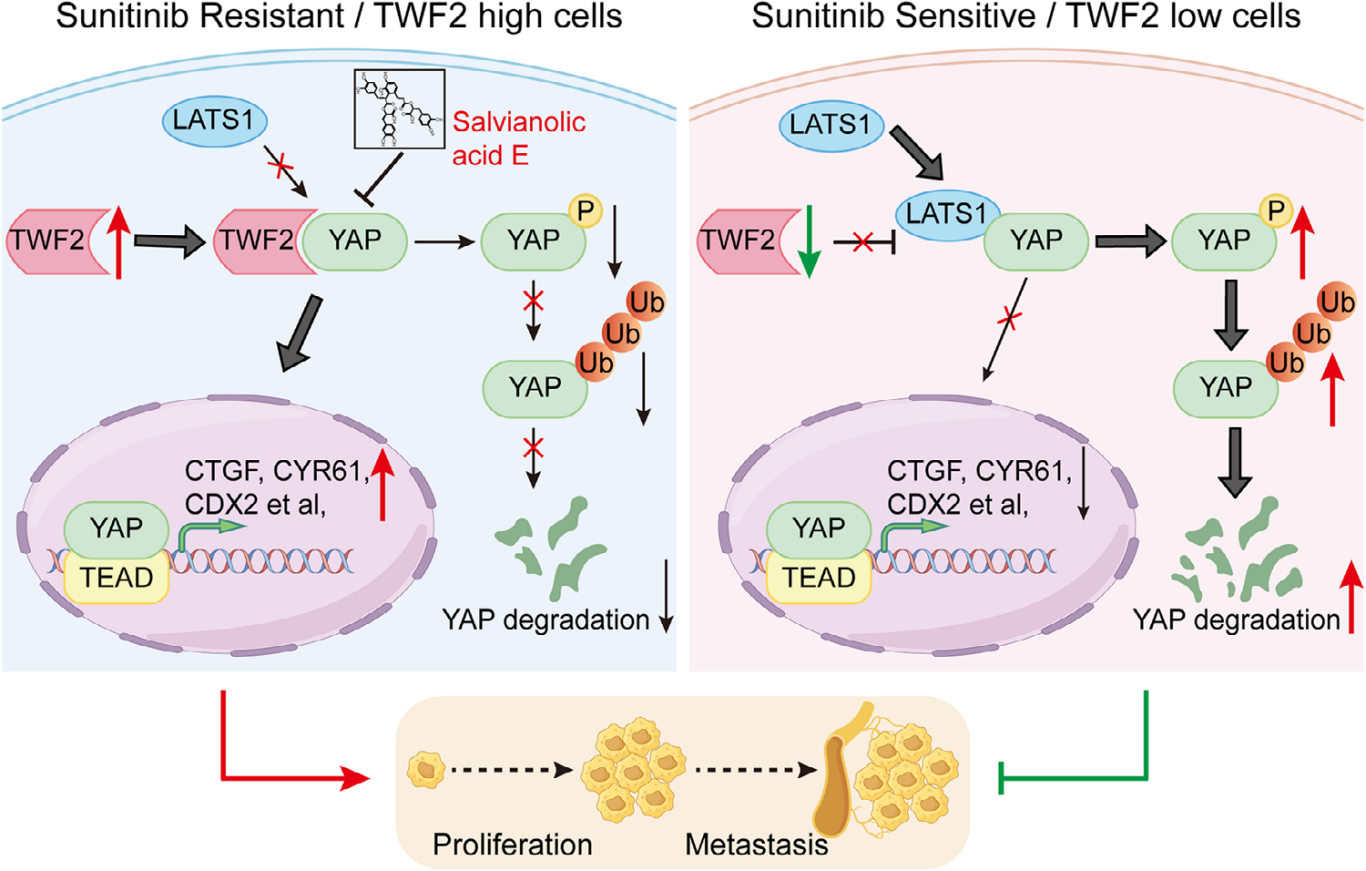
Schematic model illustrating the role of *TWF2* in sunitinib resistance and RCC progression. In sunitinib‐resistant and high grade malignant RCC cells, *TWF2* is upregulated and interacts with YAP, competing with LATS1 and thereby preventing YAP ubiquitination and degradation. Stabilized YAP translocates into the nucleus and activates transcription of target genes, promoting RCC proliferation, metastasis, and drug resistance. In sunitinib‐sensitive and low grade malignant RCC cells, reduced *TWF2* expression permits YAP phosphorylation and subsequent degradation, limiting transcriptional activity. The small‐molecule compound Sal E disrupts the *TWF2*–YAP interaction and enhances the therapeutic efficacy of sunitinib in RCC.

*TWF* is a highly conserved actin monomer‐binding protein composed of two actin‐depolymerizing factor homology domains, widely present in eukaryotes, except for plants.^[^ ^48^ ^]^ In mammals, *TWF1* and *TWF2* exhibit distinct tissue‐specific expression patterns. *TWF1* predominates in embryonic and most nonmuscle adult tissues, while *TWF2* is enriched in adult cardiac and skeletal muscles.^[^ ^19^ ^]^ Although *TWF1* has been implicated in oncogenesis, particularly in breast tumors,^[^ ^49^, ^50^ ^]^ the mechanistic contributions of *TWF2* to tumor development remain insufficiently characterized. The present study identified *TWF2* through an integrative analysis of RCC cell sequencing with poor patient prognosis. Functional assays demonstrated that *TWF2* enhanced RCC cell migration, metastasis, and resistance to therapy, suggesting its potential as a therapeutic target in RCC treatment (**Figure** **9**).

The Hippo signaling pathway is an evolutionarily conserved regulatory network that governs key biological processes, including cell proliferation and differentiation, organ growth, embryogenesis, tissue regeneration, and wound healing. Dysregulation of Hippo signaling contributes to multiple pathologies, including malignancies and disorders of the eyes, heart, lungs, kidneys, liver, and immune system.^[^ ^51^, ^52^ ^]^ RNA sequencing and functional characterization in RCC cells highlighted a central role for the Hippo pathway in *TWF2*‐mediated RCC progression and therapeutic resistance. Within this pathway, LATS1/2 kinases phosphorylate and transcriptional coactivators YAP and TAZ. Hyperactivation of YAP, a downstream effector of the pathway, has been documented in numerous cancers. Elevated YAP level accumulation and nuclear accumulation have been reported in hepatocellular carcinoma,^[^ ^53^, ^54^ ^]^ lung cancer,^[^ ^55^, ^56^ ^]^ breast cancer,^[^ ^57^ ^]^ skin cancer,^[^ ^58^ ^]^ and colorectal cancer.^[^ ^59^, ^60^, ^61^ ^]^ In RCC, *TWF2* was shown to competitively bind YAP, displacing LATS1, thereby inhibiting YAP phosphorylation and subsequent proteasomal degradation. Stabilized, unphosphorylated YAP accumulates in the nucleus, engages transcription factors such as TEAD, and activates downstream gene expression programs associated with cell migration and metastatic dissemination.

Molecular docking and Co‐IP experiments with site‐directed mutants identified *TWF2* M99 and YAP M225 as key residues mediating the *TWF2*–YAP interaction. Aiming to develop targeted therapeutic strategies, small‐molecule inhibitors were screened in silico based on the 3D structure of *TWF2*. Sal E emerged as a candidate compound with the highest docking score for the *TWF2* M99 site and demonstrated synergistic antitumor efficacy when combined with sunitinib.

Sal E, a water‐soluble phenolic acid derived from *Salvia miltiorrhiza*, represents a principal polyphenolic compound within this traditional Chinese medicinal herb. *S. miltiorrhiza*, the dried root and rhizome of *S. miltiorrhiza* Bunge, has long been employed for its blood‐activating and stasis‐resolving properties. Its constituents are broadly categorized into lipophilic compounds (e.g., tanshinones) and hydrophilic compounds (e.g., salvianolic acids). As a hydrophilic component, Sal E shares a structure similarity with other salvianolic acids such as B, C, and D, typically formed by the condensation of danshensu (3,4‐dihydroxyphenylactic acid) with various aromatic acids.^[^ ^62^ ^]^ With a molecular formula C_30_H_26_O_12_, Sal E contains multiple phenolic hydroxyl groups and a conjugated system that confer potent antioxidant activity.^[^ ^63^ ^]^ Sal E was identified as a bioactive constituent of Naoxintong Capsules. It exerts pronounced anti‐inflammatory and antioxidative effects through suppression of NF‐κB, MMP‐9, and nitric oxide expression, as well as inhibition of the PI3K/AKT signaling pathway, thereby supporting its use in the treatment of coronary heart disease.^[^ ^64^ ^]^ Despite limited direct evidence, biological activity inferred from related salvianolic acids and experimental findings indicates therapeutic potential across several domains, including cardiovascular protection, antioxidant and neuroprotection, anti‐inflammation effects, organ protection, antitumor effects, and immunomodulation.^[^ ^65^, ^66^, ^67^, ^68^, ^69^, ^70^, ^71^ ^]^ Further experimental and clinical investigations are necessary to elucidate its specific mechanisms of action and safety profile, facilitating its development for novel therapeutic applications, particularly in oncology.

In the present study, integrative multiomics analysis identified six candidate genes, *CTHRC1*, *TWF2*, *COL6A3*, *SLC38A5*, *IFI44*, and *OASL*, associated with RCC progression and therapeutic resistance. Among these, *TWF2* was prioritized for in‐depth functional validation based on previous evidence. However, the biological roles of the remaining genes in RCC pathogenesis warrant further elaboration.

*CTHRC1* encodes a conserved, secreted glycoprotein enriched in the tumor microenvironment and secreted by both tumor and stromal cells. It modulates extracellular matrix interactions and growth factor signaling, thereby supporting tumor–stromal interactions and promoting cell proliferation, migration, and invasion. Aberrant *CTHRC1* expression has been reported across several solid tumors and is correlated with poor prognosis, suggesting its potential as a therapeutic target in RCC.^[^ ^72^, ^73^, ^74^, ^75^ ^]^ *COL6A3* encodes the α3 subunit of type VI collagen, a key structural ECM protein involved in maintaining tissue integrity and regulating developmental processes. In osteosarcoma, *COL6A3* promotes malignancy, whereas in bladder cancer, its downregulation suppresses proliferation, angiogenesis, and epithelial–mesenchymal‐transition‐associated metastasis, underscoring its context‐dependent function.^[^ ^76^, ^77^, ^78^, ^79^ ^]^ *SLC38A5* functions as an amino acid transporter and plays a critical role in cancer metabolism. Dysregulated amino acid homeostasis contributes to tumor progression by maintaining an alkaline intracellular pH, facilitating cell cycle progression, and enhancing proliferative capacity. Its metabolic regulatory function highlights its importance in tumor biology.^[^ ^80^, ^81^, ^82^ ^]^ *IFI44* and *OASL* are interferon‐stimulated genes implicated in antiviral responses and immune regulation. *IFI44* and *OASL* have been associated with tumor progression and immune evasion, suggesting that they exert analogous roles in immune–tumor interactions.^[^ ^83^, ^84^, ^85^, ^86^ ^]^ While *TWF2* was the primary focus of this investigation, the remaining five genes demonstrate potential involvement in key tumor‐associated processes such as extracellular matrix remodeling, metabolic adaptation, and immune regulation. Elucidating the specific roles in RCC progression and therapeutic resistance represents an important direction for future research.

Sunitinib is a first‐line treatment agent for advanced RCC and functions as an oral multitarget receptor Tyrosine kinase inhibitors that specifically inhibits signaling pathways involving vascular endothelial growth factor and platelet‐derived growth factor receptor. Despite its clinical utility, ≈60–70% of patients with metastatic RCC exhibit intrinsic resistance to sunitinib, and even those who initially respond frequently relapse within 11 months of treatment.^[^ ^87^ ^]^ This study demonstrated that depletion of *TWF2* enhances sensitivity to sunitinib, indicating that *TWF2* may serve as a promising therapeutic target in RCC. Furthermore, the *TWF2* inhibitor Sal E was identified, and combination treatment with Sal E and sunitinib produced a synergistic antitumor effect in patient‐derived RCC PDX mice, outperforming sunitinib monotherapy. These findings suggest that combining *TWF2*‐targeting agents with sunitinib may represent a novel and effective therapeutic strategy for advanced RCC, potentially improving the efficacy of targeted treatment. Collectively, the results provide a safe and promising candidate for RCC‐targeted drug development and offer novel insights into therapeutic strategy innovation.

Although this study has substantially advanced the understanding of *TWF2*'s role in RCC progression and drug resistance and identified Sal E as a potential therapeutic compound, certain limitations remain. The analysis involved 120 patients, providing valuable insights; however, the limited sample size and demographic homogeneity constrain the generalizability of the findings. Future studies should include more diverse populations encompassing various racial, ethnic, and geographical backgrounds to enable a comprehensive assessment of *TWF2* expression and its prognostic relevance across patient subgroups. While in vitro and in vivo models provided mechanistic insights, they do not fully capture the complexity of the tumor microenvironment and host immune responses. To address this, incorporating advanced models such as genetically engineered spontaneous RCC, patient‐derived organoids, and humanized mouse systems would improve physiological relevance and translational potential. Although Sal E was identified as a *TWF2*–YAP interaction inhibitor with synergistic effects alongside sunitinib, its pharmacokinetic and pharmacodynamic profiles, including safety and toxicity, remain to be characterized. Structural optimization of Sal E to improve drug‐like properties, such as bioavailability, metabolic stability, and target selectivity, should be prioritized. Addressing these limitations through expanded sample cohorts, deeper mechanistic studies, refined preclinical models, interdisciplinary collaboration, and increased awareness will advance the understanding of RCC pathogenesis and support the development of improved diagnostic, prognostic, and therapeutic strategies for RCC management.

In conclusion, this study provides compelling evidence that *TWF2* plays a pivotal role in promoting RCC progression and sunitinib resistance by interacting with and stabilizing YAP. The combination of *TWF2*–YAP interaction inhibitor Sal E with sunitinib represents a promising therapeutic strategy to improve clinical outcomes in RCC patients. Future studies would focus on validating the clinical utility of Sal E and the broader applicability of the *TWF2*–YAP axis as a therapeutic target to optimize treatment strategies for RCC patients.

## 4 Experimental Section

### Patients and Specimens

Human ccRCC and adjacent peritumor tissue specimens were obtained from patients who underwent surgery at the Department of Urology, First Affiliated Hospital, Sun Yat‐sen University (FAH‐SYSU, Guangzhou, China). Tumor staging was conducted according to the 8th edition of the American Joint Committee on Cancer TNM system. Inclusion criteria were: 1) histological diagnosis of primary ccRCC and 2) receipt of partial or radical nephrectomy. Exclusion criteria comprised the presence of other malignancies or incomplete clinical information. This study adhered to the principles outlined in the Declaration of Helsinki. All patients provided written informed consent, and experimental protocols were approved by the Medical Ethics Committee of FAH‐SYSU. Samples for RNA analysis were preserved in RNAlater and stored at −80 °C, while tissues for western blotting were frozen at −80 °C. Clinicopathological characteristics of the 120 patients are detailed in Table S1 (Supporting Information). Tumor specimens from 20 ccRCC patients treated with sunitinib were obtained to evaluate treatment response. All patients underwent nephrectomy before sunitinib therapy, and primary tumors were analyzed. Treatment response was assessed according to the Response Evaluation Criteria in Solid Tumors, with disease control, comprising complete response, partial response, and stable disease, classified as response, and progressive disease defined as nonresponse.

### Cell Culture

The immortalized renal epithelial cell line (HK2) and human RCC cell lines (786‐O, 769‐P, A‐498, ACHN, Caki‐1, and Caki‐2) were obtained from the American Type Culture Collection. The OSRC2 RCC cell line and 293T cells were sourced from the Cell Bank of the Chinese Academy of Sciences (Shanghai, China). Sunitinib‐resistant and control 786‐O cell lines were developed in vivo based on previously published method with modification.^[^ ^87^ ^]^ A total of 5 × 10^6^ 786‐O cells were subcutaneously injected into the flanks of nude mice. Once tumor volumes reached 200 mm^3^, mice were administered oral sunitinib (40 mg kg^−1^ day^−1^; MedChem Express) or dimethyl sulfoxide (DMSO) following a regimen of 4 weeks on treatment followed by 2 weeks off. After one treatment cycle, tumors were excised and dissected into 1 mm^3^ fragments and transplanted into secondary mice, which subsequently received sunitinib or vehicle treatment. Tumors from tertiary mice were excised, mechanically dissociated, and digested in Dulbecco's Modified Eagle Medium (DMEM; Gibco, USA) supplemented with 0.2% collagenase IV, 0.01% hyaluronidase, and 0.002% DNase I. The resulting cell suspension was centrifuged at 300 × *g*, and the pellets were cultured in a six‐well plates. These sunitinib‐resistant and sunitinib‐sensitive 786‐O/769‐P cells were designated as 786‐O‐R/769‐P‐R and 786‐O‐S/769‐P‐S, respectively.

HK2 cells were cultured in Keratinocyte Serum‐Free Medium (Gibco). The 293T and ACHN cell lines were cultured in DMEM (Gibco), while Caki‐1 cells were maintained in McCoy's 5A medium (Gibco). The remaining RCC cell lines were cultured in RPMI‐1640 medium (Gibco). All media were supplemented with 10% fetal bovine serum (FBS; Gibco) and 1% penicillin–streptomycin (Bioyard Biotechnology, China). All cells were incubated at 37 °C in a humidified atmosphere containing 5% CO_2_. Cell line authentication was performed using short tandem repeat profiling, and all lines were confirmed to be free of mycoplasma contamination.

### Plasmid, RNA Interference, and Lentivirus Construction

Short hairpin RNA (shRNA) targeting *TWF2* and YAP, along with a scrambled control shRNA, was obtained from HanYi Biosciences Inc. (China). Target sequences are presented in Table S2 (Supporting Information). Recombinant plasmids for *TWF2* overexpression, as well as empty vector controls and plasmids encoding *YAP* and *LATS1*, were synthesized and constructed by the same company. A ubiquitin‐expressing plasmid was generously provided by Prof. Jianping Guo (FAH‐SYSU, Guangzhou, China). Truncation and point mutation plasmids for *TWF2* and *YAP* were synthesized by HanYi Biosciences Inc., and the corresponding primer sequences are listed in Table S3 (Supporting Information). All plasmids were sequence verified prior to transfection. Lentiviral packaging and negative control plasmids were acquired from HanYi Biosciences Inc. 293T cells were transfected with *TWF2* overexpression plasmids using Lipofectamine 3000 (#L3000015, Invitrogen). After 48 h, supernatants containing *TWF2*‐overexpressing lentivirus were harvested and applied to infect 786‐O and 769‐P cells. Stably transduced lines were selected using puromycin (5 µg mL^−1^), and overexpression was quantified using RT‐qPCR.

### RT‐qPCR Analyses

Total RNA was extracted from cell lines or tissue samples using the EZ‐press RNA Purification Kit (EZBioscience, USA). RNA concentration was quantified with a NanoDrop 2000 spectrometer. First‐strand complementary DNA (cDNA) was synthesized from 1 µg total RNA using the 4 × Reverse Transcription Master Mix (EZBioscience, USA) according to the manufacturer's instructions. RT‐qPCR was conducted using 2 × SYBR Green qPCR Master Mix (EZBioscience, USA) on the Applied Biosystems QuantStudio 5 Real‐Time PCR System. Primer sequences used in this study are provided in Table S4 (Supporting Information).

### Western Blotting

Cells were lysed in RIPA lysis buffer (#P0013, Beyotime, China) supplemented with a protease inhibitor cocktail (CoWin Biosciences, China) on ice for 15 min. Protein concentration was determined using the Pierce BCA Protein Assay Kit (ThermoFisher, USA) and measured at 562 nm. Equal amounts of protein were denatured in 1 × SDS loading buffer, resolved using SDS‐PAGE, and transferred to 0.45 µm polyvinylidene difluoride membranes (Merck Millipore, Billerica, MA, USA). Membranes were blocked and incubated with primary antibodies at 4 °C for over 12 h. The primary antibodies for western blotting were as follows: *TWF2* (#ab189828, Abcam), YAP (#14074, CST), phospho‐YAP (Ser61) (#75784, CST), phospho‐YAP (Ser109) (#53749, CST), phospho‐YAP (Ser127) (#13008, CST), phospho‐YAP (Ser381) (#13619, CST), LATS1 (#66569‐1‐Ig, Proteintech), LATS2 (#20276‐1‐AP, Proteintech), *CTGF* (#10915‐1‐AP, Proteintech), alpha tubulin (#11224‐1‐AP, Proteintech), GAPDH (#60004‐I‐Ig, Proteintech), H3 (#ab1791, Abcam), anti‐Flag (#F7425, Sigma‐Aldrich), anti‐His (#ab18184, Abcam), and anti‐HA (#ab236632, Abcam). Following incubation with HRP‐conjugated anti‐rabbit or mouse IgG secondary antibodies (Proteintech) for 1 h at room temperature, protein bands were visualized using chemiluminescence (Tanon).

### Cell Proliferation and Colony Formation Assays

Cell proliferation was measured using the CCK‐8 (Dojindo, Tokyo, Japan), according to the manufacturer's instructions. A total of 1500 cells were seeded per well in 96‐well plate. After incubation, 10 µL of CCK‐8 solution was added to each well and incubated for 2 h at 37 °C. Optical density was measured at 450 nm using a spectrophotometer. All experiments were performed in triplicates. For IC50 determination, cells were treated with graded concentrations of sunitinib for 48 h, and IC50 values were calculated using GraphPad Prism (v9.0) (GraphPad Software). For colony formation assays, 1000 cells were seeded in 6‐well plates containing 2 mL of complete medium and cultured for 2 weeks. Colonies were fixed with 4% paraformaldehyde (PFA) for 20 min at room temperature and stained with 0.4% crystal violet. Colony numbers were quantified using ImageJ software.

### Wound Healing and Transwell Assays

Wound healing assays were performed by seeding cells in 6‐well plates in triplicate. Following full confluence, a scratch was introduced using a pipette tip. Wound closure was monitored at 0 and 24 h postscratch. Transwell assays were performed to evaluate cell migration and invasion. A total of 5 × 10^4^ 786‐O or 769‐P cells were suspended in 200 µL of serum‐free RPMI 1640 medium and placed in the upper chamber of 24‐well transwell inserts (Corning, NY, USA). For invasion assays, membranes were precoated with Matrigel (Corning, NY, USA) at 37 °C for 1 h. The lower chambers were supplemented with RPMI 1640 medium containing 10% FBS as a chemoattractant. After incubation (8 h for migration, and 16 h for the invasion), cells on the lower surface were fixed with 4% PFA (Beyotime, China) for 30 min and stained with 0.4% crystal violet (Beyotime, China) for 30 min and counted under a microscope.

### Co‐IP Assay

Cells were transfected with the indicated plasmids and lysed in a cell lysis buffer (Beyotime) supplemented with 1× protease inhibitor cocktail (CoWin Biosciences) on ice for 30 min. 10% of each lysate was collected as the input controls and stored at −80 °C. For immunoprecipitation of YAP‐binding proteins, cell lysates were incubated with protein A/G magnetic beads (Thermo Scientific) for 2 h, followed by overnight incubation with anti‐YAP antibody (#14074, CST) at 4 °C. After washing the beads, bound proteins were denatured with 1 × SDS loading buffer and subjected to SDS‐PAGE. For immunoprecipitation of *TWF2*‐Flag‐tagged proteins, lysates were incubated overnight at 4 °C with anti‐Flag magnetic beads (#B26101, Selleck). Beads were washed 5 times, and conjugated proteins were eluted with 400 µg mL^−1^ Flag peptide (RP10586, GenScript) for subsequent silver staining or LC–MS/MS analysis (Wininnovate Bio, China). Additional Co‐IP experiments followed the same protocol, using anti‐HA magnetic beads (#B26201, Selleck) or protein A/G magnetic beads (#B23201, Selleck) as appropriate for the tag or antibody use.

### Hematoxylin–Eosin (H&E) and Immunohistochemistry (IHC) Staining

H&E and IHC staining were performed on 4 µm paraffin‐embedded tissue sections. Slides were dewaxed, rehydrated, and processed accordingly. H&E staining was conducted using hematoxylin and eosin (E607318, Sangon Biotech) for 3 min. For IHC, sections underwent heat‐induced epitope retrieval followed by blocking with 20% goat serum for 30 min. Primary antibodies for IHC staining were used to incubate with the tissues overnight at 4 °C. The primary antibodies used were *TWF2* (#ab189828, Abcam) and YAP (#14074, CST). HRP‐conjugated secondary antibody (K5007, Dako) was incubated for 30 min on the following day. Visualization was performed using a DAB Kit (K5007, Dako), followed by hematoxylin counterstaining. Staining index (SI) was independently assessed by two pathologists. Staining intensity was scored as follows: 0 = negative, 1 = weak, 2 = intermediate, and 3 = strong. The proportion of positive cells was classified as: 0 = 5%, 1 = 5–25%, 2 = 26–50%, 3 = 51–75%, and 4 = 76–100%. The SI was calculated using the formula: SI = staining intensity (0–3) × proportion of positive cells (0–4).

### Immunofluorescence Staining

Cells were seeded in confocal dishes and cultured for 24 h. Following phosphate‐buffered saline (PBS) washes, cells were fixed with 4% PFA for 15 min at room temperature and permeabilized with 0.5% Triton X‐100 for an additional 15 min. Blocking was performed using 5% Bovine Serum Albumin (Sigma‐Aldrich) for 1 h. Primary antibodies targeting *TWF2* (#ab189828, Abcam) or YAP (#14 074, CST,) were applied and incubated overnight at 4 °C. After washing, Alexa‐Fluor‐conjugated secondary antibodies (Invitrogen) were added for 1 h at room temperature. Nuclear staining was performed using 4,6‐diamidino‐2‐phenylindole (D3571, Invitrogen). Imaging was conducted with an OLYMPUS FV3000 confocal microscope (Olympus).

### Flow Cytometry

Tumor cells were detached using trypsin and washed with PBS supplemented with 1% FBS. Apoptosis rate analysis was performed using the Annexin V–APC/PI apoptosis detection kit (AT107, Multi Sciences), according to the manufacturer's instructions. A total of 1 × 10^6^ cells were resuspended in 1 × binding buffer, followed by incubation with 5 µL Annexin V–APC and 10 µL propidium iodide (FXP023, 4A Biotech, China) for 5 min at room temperature. Samples were analyzed using a CytoFLEX Flow cytometer (Beckman Coulter, USA), and data interpretation was performed with FlowJo v10 software.

### RNA‐Sequencing Analysis

Total RNA was extracted from shCtrl and sh*TWF2* 769‐P cells using TRIzol reagent (Invitrogen), according to the manufacturer's instructions. The mRNA was enriched by depleting ribosomal RNA, digested, and reverse‐transcribed into second‐strand cDNA. A cDNA library was constructed and sequenced by Tsingke Biotechnology (China). High‐quality raw sequencing reads were mapped to the human reference genome (GRCh38) using Hisat2 alignment tool. Gene expression level was normalized to fragments per kilobase of transcript per million mapped reads (FPKM). Differentially expressed genes were identified using the DESeq2 package.

### Cycloheximide Chase Assays

Cells were treated with 20 µg mL^−1^ CHX (#HY‐12320, MedChemExpress) for 0, 4, 8, 12, and 24 h. Protein levels were assessed via western blot analysis. Band intensities were quantified using ImageJ software (National Institutes of Health, Bethesda, MD, USA).

### Cell Fractionation

ccRCC cells were harvested, washed, and resuspended in cold PBS. Cytoplasmic and nuclear protein fractions were separated using a Nuclear and Cytoplasmic Protein Extraction Kit (#P0028, Beyotime) according to the manufacturer's protocol. Protein concentrations were quantified using a BCA protein assay kit, and fractions were subsequently analyzed via western blotting.

### Ubiquitination Assay

The ccRCC cells were transfected with His‐ubiquitin for 48 h, and the cells were harvested and resuspended in buffer A (6 m guanidine‐HCl, 0.1 mol L^−1^ Na_2_HPO_4_, 0.1 mol L^−1^ NaH_2_PO_4_, 10 mm imidazole, pH 8.0). The cells were then lysed via sonication and incubated with protein A/G magnetic beads (Thermo Scientific) for 2 h and subsequently incubated with anti‐YAP antibody (#14074, CST) overnight at 4 °C. The pull‐down products were washed once with buffer A, once with 1:3 buffer A/buffer TI (25 mm Tris‐HCl and 20 mm imidazole, pH 6.8), and twice with buffer TI. His‐Ub‐conjugated proteins pulled down by the beads were analyzed using western blotting.

### Virtual Screening of *TWF2*–YAP Interaction Inhibitors

The 3D structure of *TWF2* was predicted using AlphaFold and processed using the Receptor Grid Generation module. The grid file was generated at the center of the M99 site. A total of 76 331 compounds obtained from four commercial chemical libraries (Bioactive Compound Library, Asinex Macrocycles Library, Protein–Protein Interaction Library, and Protein–Protein Interaction Inhibitor Library) were subjected to virtual screening. Molecular docking was performed using Schrödinger's Glide docking module (HTVS, SP, XP). The top ten‐ranked compounds by binding affinity score were selected for experimental validation. Molecular docking structures were visualized using PyMOL (The PyMOL Molecular Graphics System, Schrödinger, LLC).

### BLI

BLI assays were performed using the Octet R8 system (OCTET‐R8, Sartorius). HIS1K biosensors (18‐5120, Sartorius) were pre‐equilibrated for 10 min in buffer containing 20 mm *N*‐(2‐hydroxyethyl)piperazine‐29‐(2‐ethane‐sulfonic acid) (HEPES) (pH 7.5), 300 mm NaCl, 2 mm MgCl_2_, and 0.02% Tween. His_6_‐tagged YAP was immobilized onto HIS1K biosensor tips, followed by exposure to serial dilutions of *TWF2* protein. Each cycle included a 120 s baseline, a 360 s association phase, and a 360 s dissociation phase. Data were analyzed using Octet BLI Discovery software (v12.2), and the *K*_d_ was calculated accordingly.

### ITC Assay

Wild‐type Flag‐*TWF2* and M99A mutant Flag‐*TWF2* proteins were overexpressed in 293F cells and purified using anti‐Flag magnetic beads. Binding of *TWF2* to Sal E was assessed using a MicroCal ITC200 (Malvern) at 25 °C in buffer containing 20 mm HEPES (pH 7.5), 300 mm NaCl, and 2 mm MgCl_2_. A total of 1 mm Sal E was titrated against 100 µm *TWF2* protein in 2 µL increments. Resulting heat changes from each injection were integrated and the values were fitted to a standard single‐site binding model analyzed using the PEAQ‐ITC program provided by the manufacturer.

### In Vivo Mouse Experiments

All animal experiments were approved by the Institutional Animal Care and Use Committee and the Animal Ethics Committee of SYSU and conducted in accordance with institutional and national guidelines. Four weeks old male BALB/c nude mice and six weeks old immunodeficient NCG mice (NOD/ShiLtJGpt‐Prkdc^em26Cd52^Il2rg^em26Cd22^/Gpt) were purchased from GemPharmatech (Nanjing, Jiangsu, China) and maintained under specific pathogen‐free conditions. Mice were randomly assigned to experimental groups. For subcutaneous xenograft model, 5 × 10^6^ ccRCC cells were injected into the flanks of BALB/c nude mice. Tumor size was measured weekly using the formula: tumor volume = 0.5 × length × width^2^. After seven weeks, tumors excised, weighed, and processed for histological analyses. Pulmonary metastasis assays involved tail vein injection of 5 × 10^6^ cells. After 8 weeks, mice were euthanized, and lung metastases were quantified and confirmed using H&E staining

For PDX models, fresh ccRCC tissue specimens from patients at FAH‐SYSU were obtained with informed consent and institutional approval. Tumor tissues were minced into 1–2 mm^3^ fragments and subcutaneously implanted into NCG mice. Once the tumors reached ≈100 mm^3^, they were serially passaged to establish stable PDX lines. For treatment, mice bearing ≈100 mm^3^ tumors were randomized into four groups: vehicle, sunitinib, Sal E, and Sal E plus sunitinib. Sal E (10 mg kg^−1^) was administered intraperitoneally every other day for 4 weeks, while sunitinib (40 mg kg^−1^ per day) was delivered orally using a 4 weeks on/2 weeks off regimen. Mice were subsequently sacrificed, and tumors were excised for further analysis.

### Bioinformatics Analysis

Gene expression profiles and clinical data for patients diagnosed with ccRCC were obtained from TCGA database (http://cancergenome.nih.gov/). A total of 539 ccRCC and 72 normal kidney samples, including HTSeq‐FPKM transcriptional data and clinical annotations, were included. Clinical and pathological characteristics of the patients are summarized in Table S5 (Supporting Information). FPKM values were converted to transcripts per kilobase million for downstream analysis. Stage‐specific expression levels were assessed using UALCAN (http://ualcan.path.uab.edu/). GSEA was performed using GSEA software (version 4.1.0; Broad Institute, USA).

### Statistical Analysis

Statistical analyses were conducted using R software (v4.1.1), SPSS software (v22.0), and GraphPad Prism (v9.0). Data were expressed as means ± standard deviation (SD). All in vitro experiments were repeated independently at least 3 times. Data distribution was evaluated for normality prior to analysis. Differences between the two groups were analyzed using a two‐tailed Student's *t*‐test. One‐way analysis of variance (ANOVA) followed by Tukey's post‐hoc test was applied for comparisons involving more than two groups. OS was defined as the time from surgery to death from any cause, and survival curves were plotted using the Kaplan–Meier method and compared using log‐rank tests. Correlation between variables was assessed using Pearson's coefficient for continuous variables and Spearman's coefficient for categorical or ranked data. Statistical significance was defined as *p* < 0.05 and denoted as follows: ns (no significance), * (*p* < 0.05), ** (*p* < 0.01), *** (*p* < 0.001).

## Conflict of Interest

The authors declare no conflict of interest.

## Author Contributions

L.F., W.L., Y.T., H.L., and K.Y. contributed equally to this work. J.Y., J.L., and L.L. conceived and supervised the project. L.F., W.L., Y.T., and H.L. performed most of the wet laboratory experiments. X.Z., M.L., Y.C., and S.L. performed bioinformatic analysis. K.Y., K.H., A.Y., Z.F., W.C., and Y.P. provided clinical samples and generated PDX models. M.C., J.W., H.F., J.C., B.G., and Z.Z. helped with biochemical experiments. L.Z., G.S., and J.L. helped with the animal experiment. L.F. performed histological assessments. L.F., J.Y., J.L., and L.L. wrote the paper. L.F., W.L., Y.T., J.Y., J.L., and L.L. revised and edited the paper. All authors discussed and approved the paper.

## Supporting information



Supporting Information

Supplemental TableS1‐S5

Supporting Information

## Data Availability

The data that support the findings of this study are available from the corresponding author upon reasonable request.
