# Supplementary material for: TWF2 Drives Tumor Progression and Sunitinib Resistance in Renal Cell Carcinoma through Hippo Signaling Suppression
Source: Adv Sci (Weinh). 2025 Sep 15;12(44):e06367. doi: 10.1002/advs.202506367 (PMC12667553; doi:10.1002/advs.202506367)
Supplement: Supplementary file 1 — Supporting Information [file ADVS-12-e06367-s002.docx]

**Supporting figures and legends**


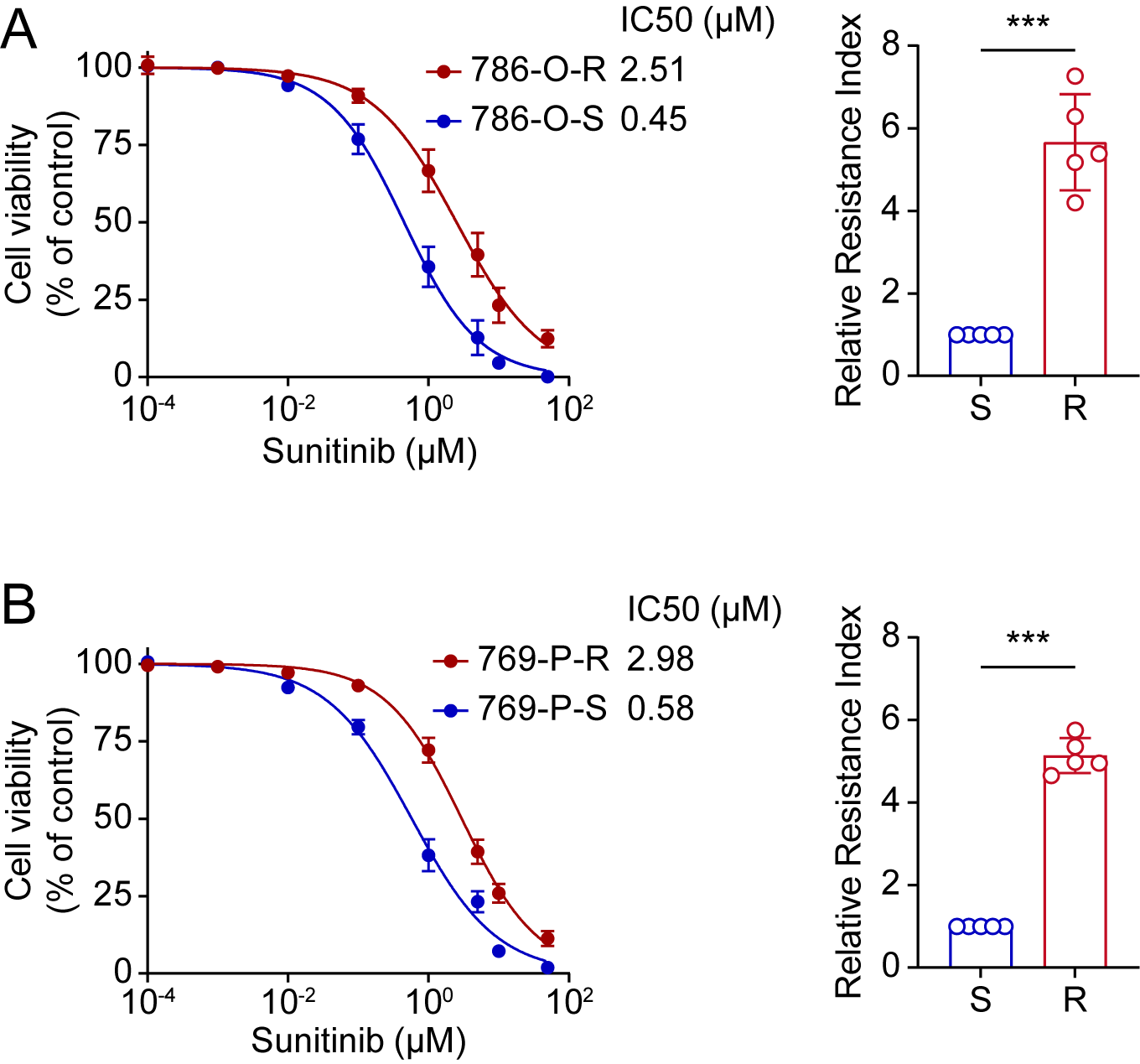


**Figure S1.** Generation of sunitinib-resistant 786-O and 769-P cells. A) Relative cell viability (left) and resistance index (right) of 786-O-R and 786-O-S cells following treatment with the indicated concentrations of sunitinib, as determined using Cell Counting Kit-8 (CCK-8) assay. B) Relative cell viability (left) and resistance index (right) of 769-P-R and 769-P-S cells under sunitinib treatment at the indicated concentrations, assessed using CCK-8. 786-O-R: sunitinib-resistant 786-O cells; 786-O-S: sunitinib-sensitive 786-O cells; 769-P-R: sunitinib-resistant 769-P cells; 769-P-S: sunitinib-sensitive 769-P cells.


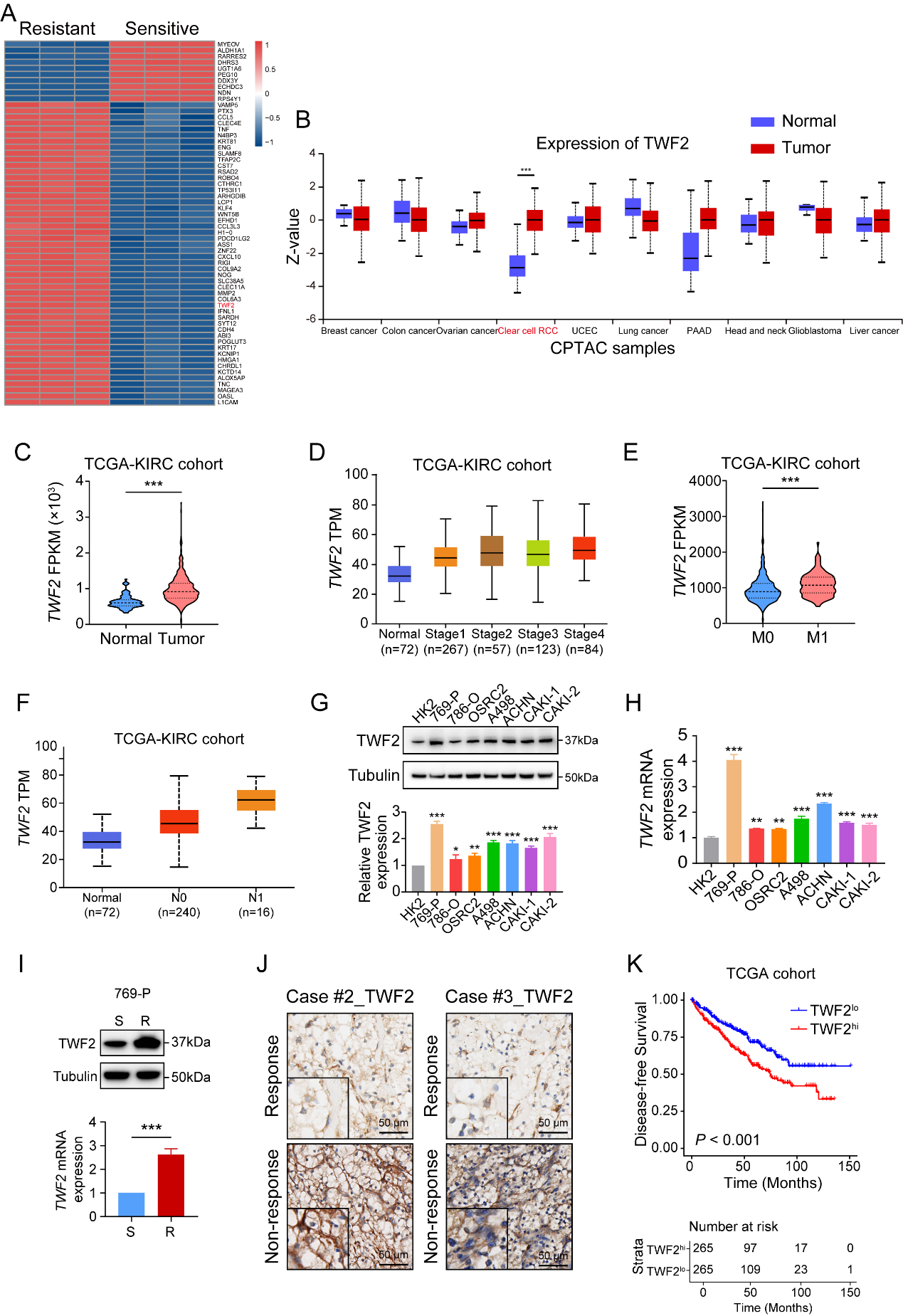


**Figure S2.** TWF2 is highly expressed in RCC and correlates with tumor malignancy, drug resistance, and poor prognosis. A) Heatmap generated from transcriptomic sequencing comparing gene expression profiles between sunitinib-resistant and sunitinib-sensitive 786-O cell. Blue indicates low expression; red indicates high expression. TWF2 are highlighted in red. B) TWF2 protein levels in ccRCC tumors and normal tissues from the CPTAC database. C) *TWF2* mRNA levels in ccRCC tumors and normal tissues from TCGA database. D) *TWF2* mRNA expression across different pathological stages in TCGA-KIRC dataset. E) *TWF2* mRNA expression stratified by metastasis status in TCGA-KIRC dataset. M0, without metastasis; M1, with metastasis. F) *TWF2* mRNA expression according to nodal metastasis status in TCGA-KIRC dataset. N0, no regional lymph node metastasis; N1, metastases in 1 –3 regional lymph nodes. G) Representative western blot (upper) and statistical analysis (bottom) of TWF2 protein expression in HK2 and RCC cell lines. Tubulin served as a loading control. H) Relative *TWF2* mRNA expression in HK2 and RCC cell lines. I) Representative western blot (upper) and relative mRNA expression levels (bottom) of TWF2 in sunitinib-sensitive (S) and -resistant (R) 769-P cells. J) Representative immunohistochemical (IHC) images showing TWF2 expression in ccRCC tissues from sunitinib-responsive and non-responsive patients. K) Disease-free survival (DFS) of patients with RCC exhibiting low (n = 265) or high (n = 265) TWF2 expression in TCGA-KIRC cohort. Data are presented as means ± SD and are analyzed by Student’s *t-*test (B, C, E, G, H, I) or log-rank test (K). **P* < 0.05; ***P* < 0.01; ****P* < 0.001.


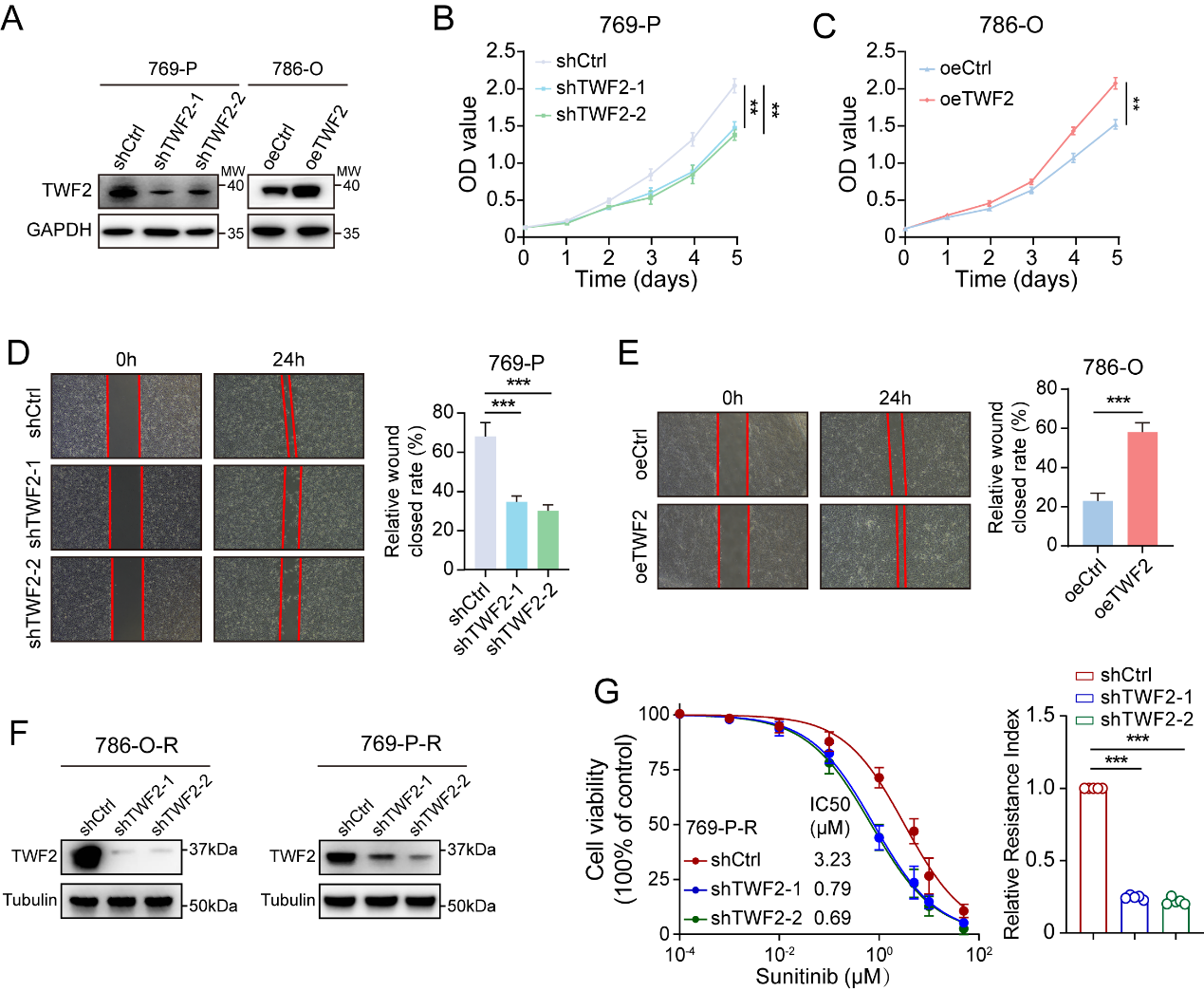


**Figure S3.** TWF2 promotes RCC progression and sunitinib resistance *in vitro.* A) Representative western blot showing TWF2 knockdown in 769-P cells and TWF2 overexpression in 786-O cells. B,C) CCK-8 assays showing proliferation of TWF2 knockdown 769-P (B), TWF2-overexpressing 786-O (C), and corresponding control cells. D,E) Wound healing assays showing the migration rates of TWF2-knockdown 769-P (D), TWF2-overexpressing 786-O (E), and corresponding control cells. F) Representative western blotting showing TWF2 knockdown in 786-O-R and 769-P-R cells. G) Relative cell viability (left) and relative resistance index values (right) for TWF2-knockdown and control 769-P-R cells treated with sunitinib, calculated using CCK-8. Data are presented as means ± SD and are analyzed by Student’s *t*-test (D, E, G) or one-way ANOVA (B, C). ***P* < 0.01; ****P* < 0.001.


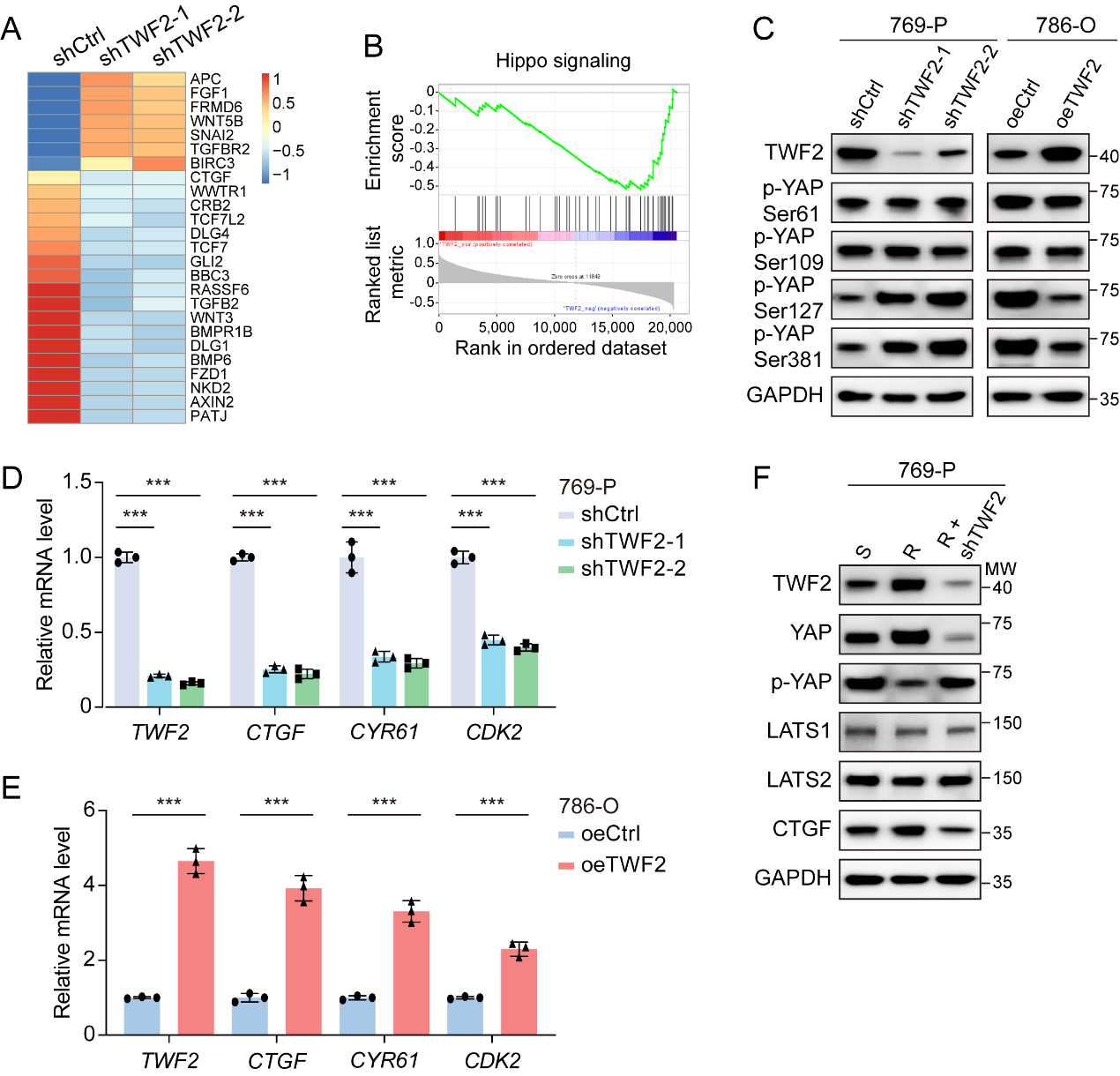


**Figure S4.** TWF2 is negatively associated with Hippo signaling. A) Heat map showing differentially expressed genes associated with the Hippo signaling pathway following TWF2 knockdown in 769-P cells. B) GSEA analysis showing that TWF2 expression is negatively associated with Hippo signaling. C) Western blot analysis showing the effects of TWF2 on the phosphorylation status of YAP at the indicated sites. D,E) RT-qPCR analysis of mRNA levels of YAP target genes in TWF2-knockdown 769-P (D), TWF2-overexpressing 786-O (E), and corresponding control cells. F) Western blot showing the expression of Hippo signaling components in the indicated 769-P cells. S: sunitinib sensitive; R: sunitinib resistant; R + shTWF2: sunitinib resistant cells with TWF2 knockdown. Data are presented as means ± SD and are analyzed by Student’s *t*-test (D, E). ****P* < 0.001.


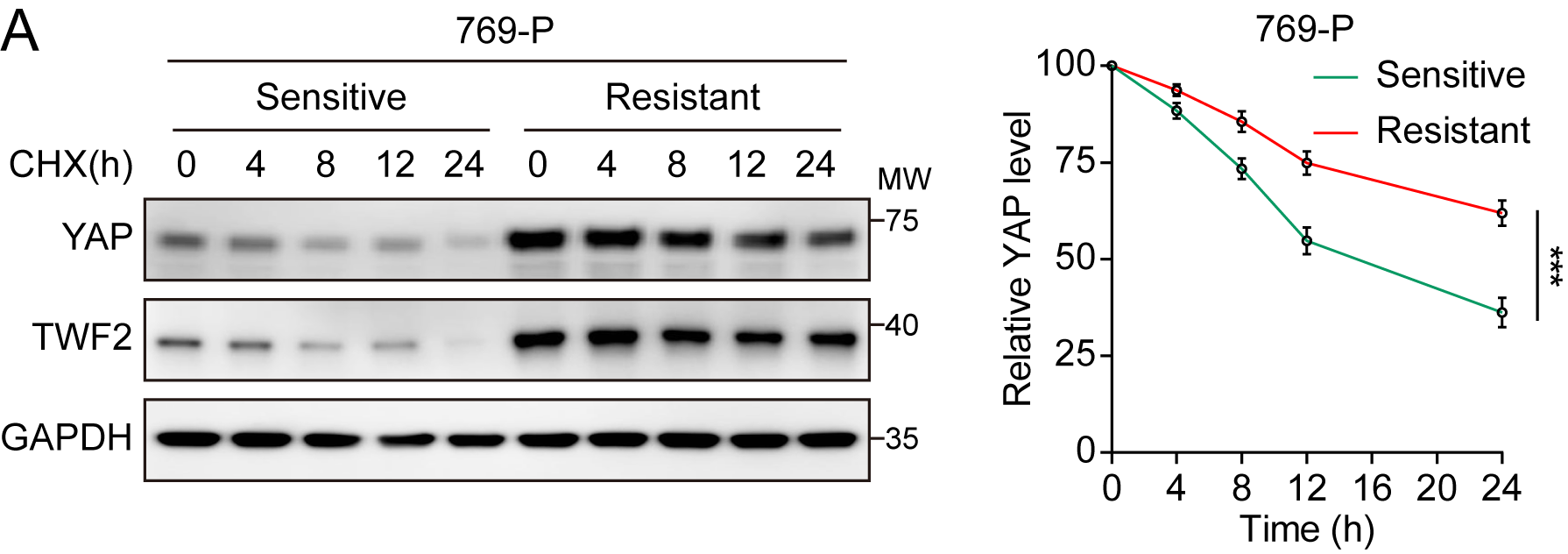


**Figure S5.** YAP degradation is delayed in sunitinib resistant cells. A) Western blot showing YAP protein stability in sunitinib-sensitive and -resistant 769-P cells following treatment with 20 μg/mL CHX for the indicated durations. Quantitative analysis is shown on the right. Data are presented as means ± SD and are analyzed by one-way ANOVA (A). ****P* < 0.001.


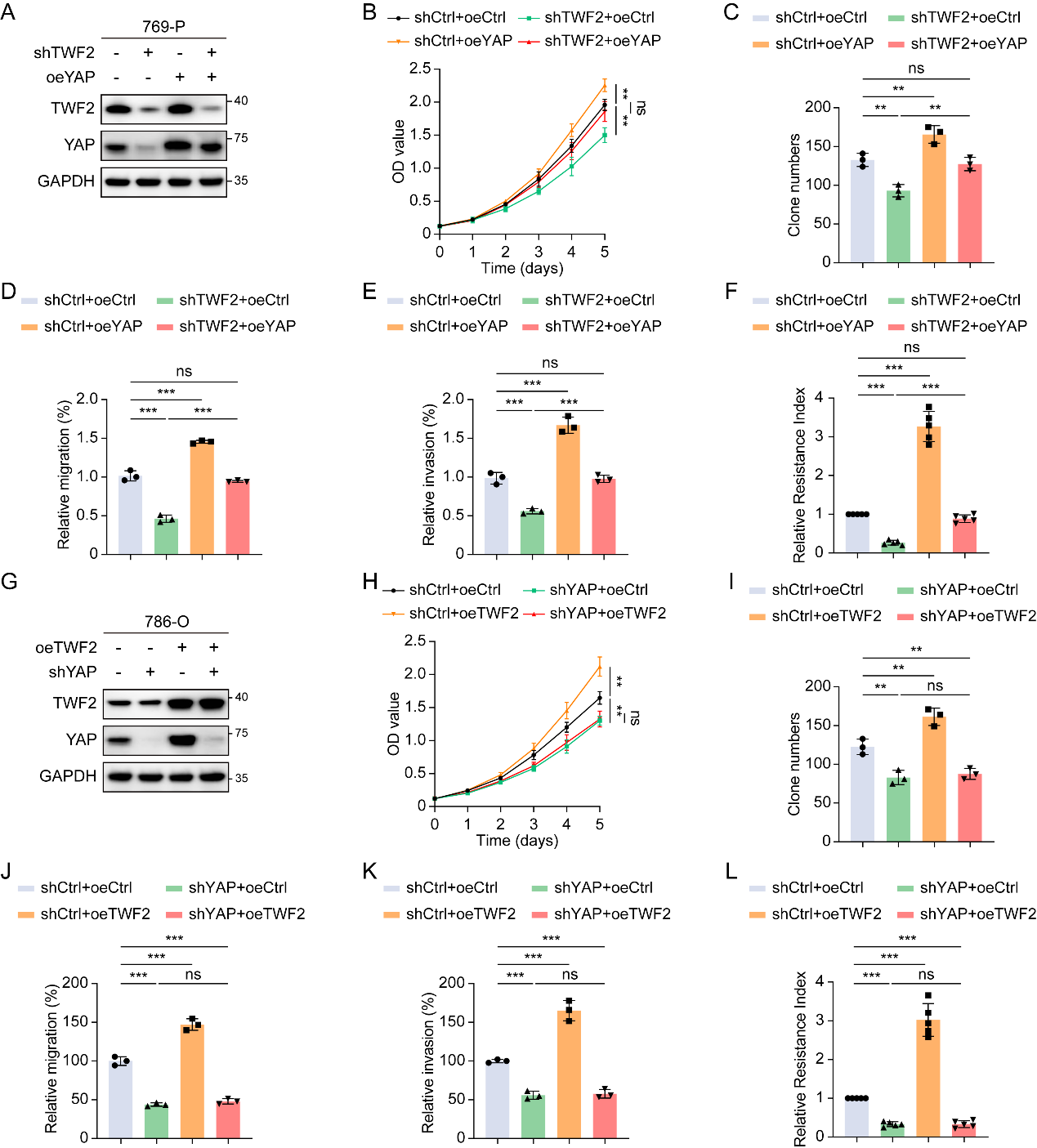


**Figure S6.** YAP is the key downstream effector of TWF2. A) Representative western blot confirming TWF2 knockdown, YAP overexpression, and combined TWF2 knockdown with YAP overexpression in 769-P cells. B-E) Proliferation (B), colony formation (C), migration (D), and invasion (E) capacities of the indicated 769-P cells. F) Relative resistance index of the indicated 769-P cells following sunitinib treatment. G) Representative western blot confirming YAP knockdown, TWF2 overexpression, and combined YAP knockdown with TWF2 overexpression in 786-O cells. H-K) Proliferation (H), colony formation (I), migration (J), and invasion (K) capacities of the indicated 786-O cells. L) Relative resistance index of the indicated 786-O cells following sunitinib treatment. Data are reported as means ± SD and are analyzed by Student’s *t*-test (C, D, E, F, I, J, K, L) or one-way ANOVA (B, H). ns, no significance; ***P* < 0.01; ****P* < 0.001.


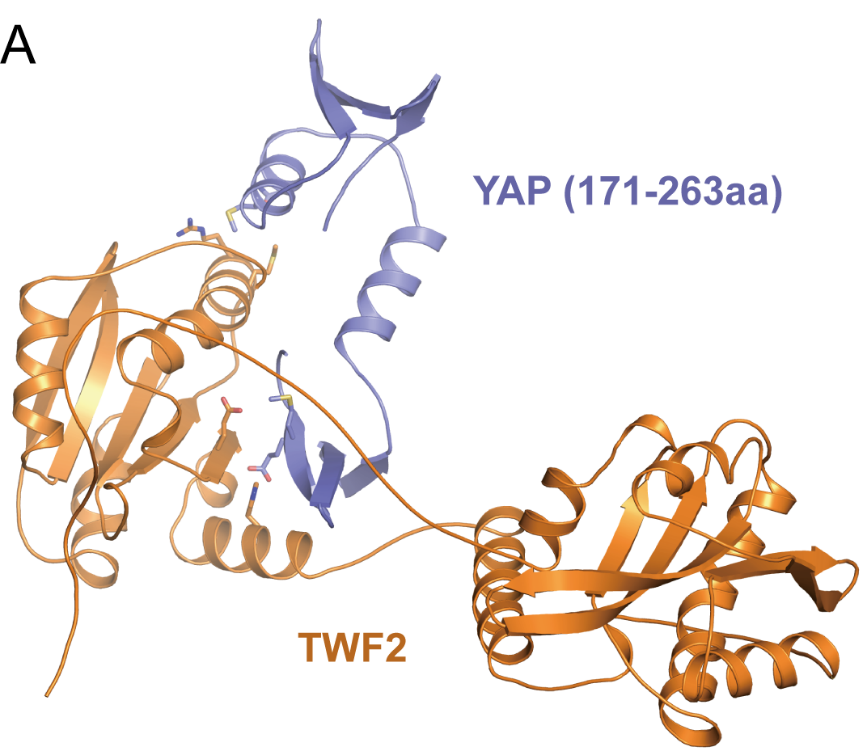


**Figure S7.** TWF2 interacts with YAP. A) Predicted structural model illustrating the interaction between TWF2 and YAP WW domain.


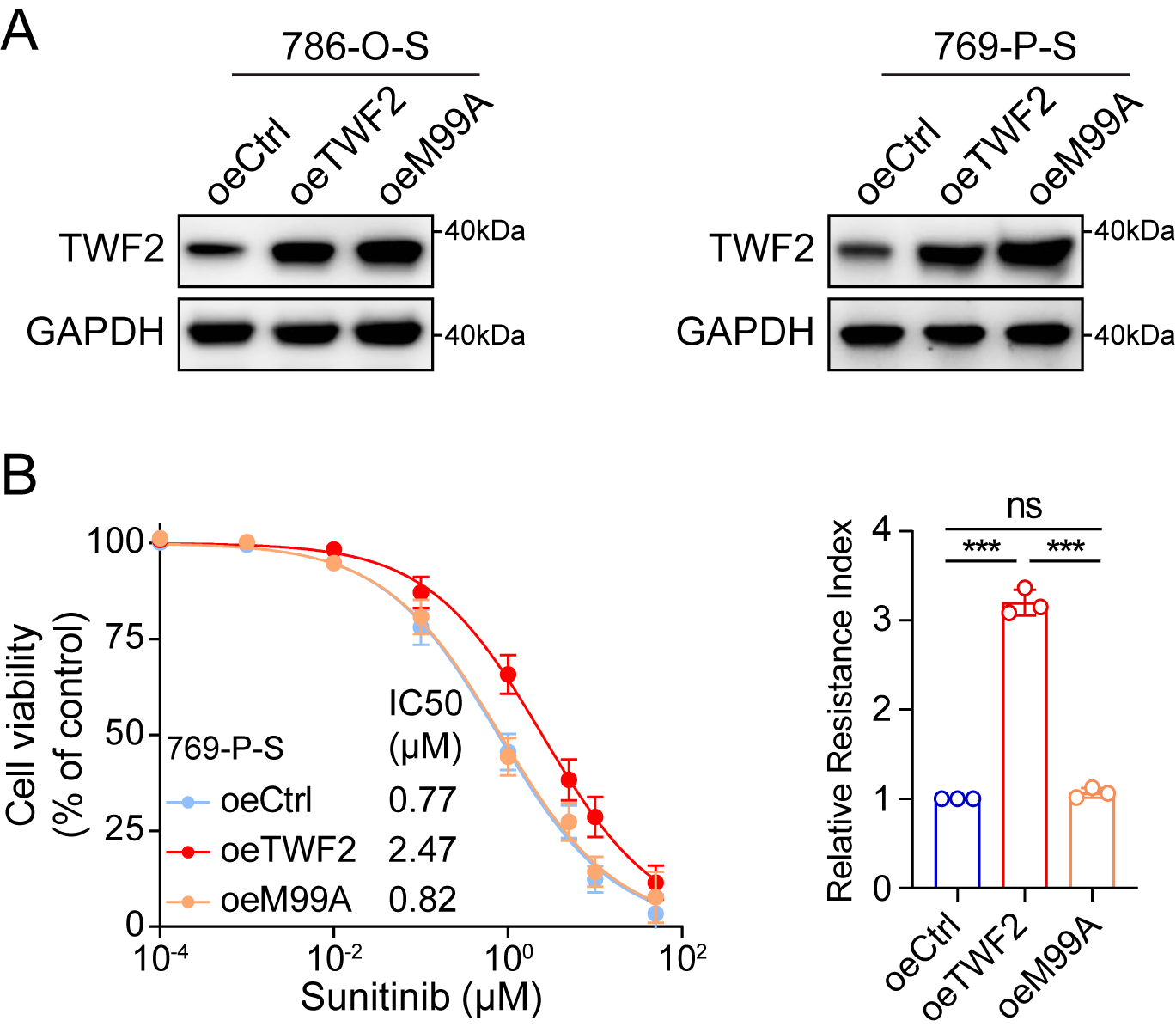


**Figure S8.** TWF2 M99A is critical for sunitinib resistance in RCC cells. A) Representative western blot showing overexpression of wild-type (WT) TWF2 or M99A-mutant TWF2 in 786-O-S (left) and 769-P-S (right) cells. B) Relative cell viability (left) and relative resistance index (right) of 769-P-S cells overexpressing wild-type or M99A-mutant TWF2 following sunitinib treatment, as determined using CCK-8 assay. Data are presented as means ± SD and are analyzed by Student’s *t*-test (B). ns, no significance; ****P* < 0.001.


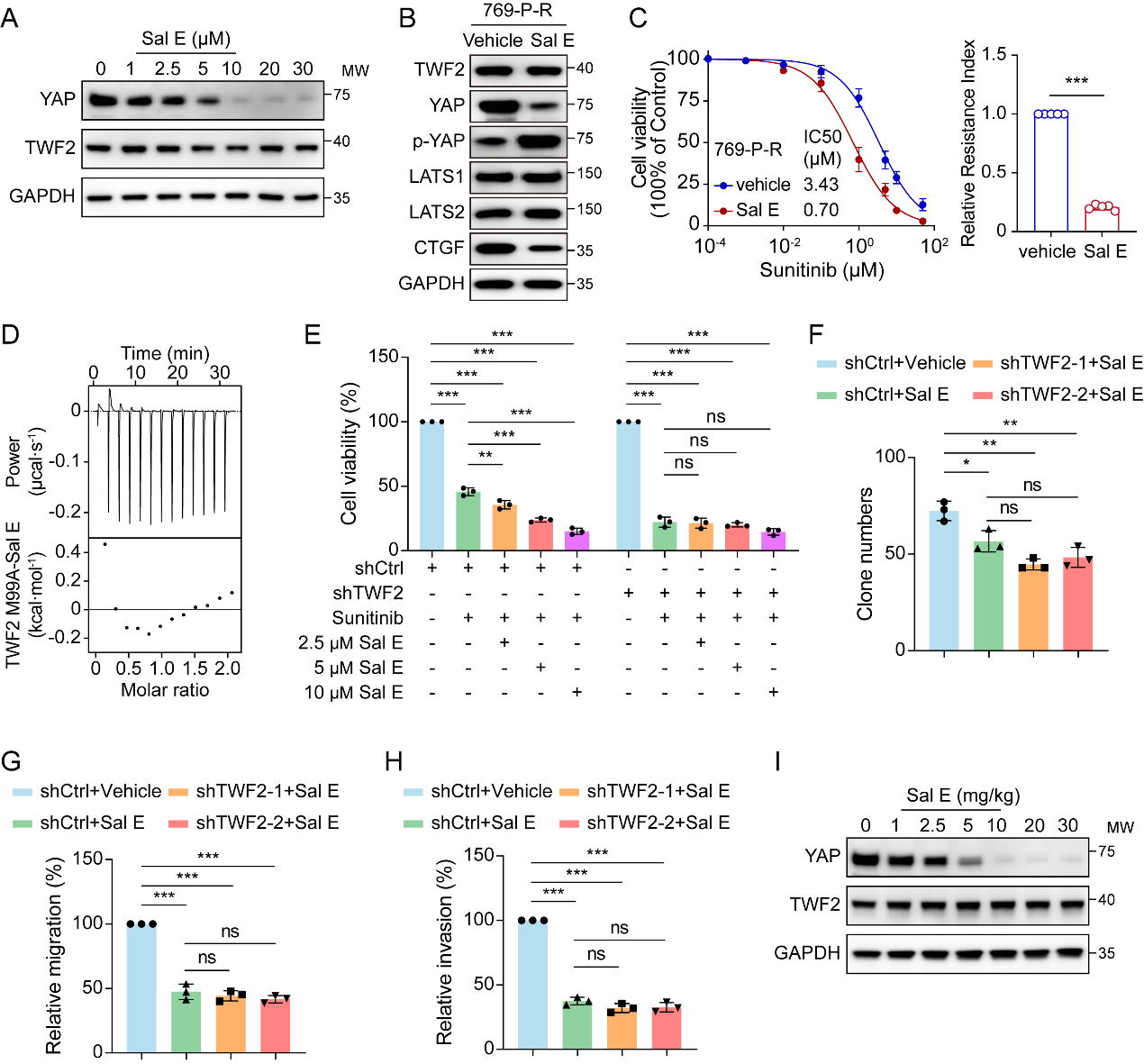


**Figure S9.** Sal E blocked the TWF2-YAP interaction and inhibited RCC cell proliferation, migration, invasion, and sunitinib resistance. A) Western blot analysis of YAP protein levels in 786-O-R cells treated with the indicated concentration of Sal E. B) Protein expression levels of Hippo signaling components in 769-P-R cells treated with 10 μM Sal E. C) Relative cell viability (left) and resistance index (right) of 769-P-R cells treated with Sal E or vehicle, in combination with sunitinib, assessed using CCK-8 assay. D) ITC assay evaluating the binding between Sal E and TWF2 M99A. E) Cell viability of control and TWF2-knockdown 786-O-R cells with indicated treatment measured using the CCK-8 assay. F-H) Colony formation (F), migration (G), and invasion (H) assays of control and TWF2-knockdown 769-P cells treated with Sal E or vehicle. I) Western blot analysis of YAP protein levels in PDX tumors treated with the indicated concentration of Sal E. Data are presented as means ± SD and are analyzed by Student’s *t*-test (C, E, F, G, H). ns, no significance; **P* < 0.05; ***P* < 0.01; ****P* < 0.001.
