# Supplementary material for: TWF2 Drives Tumor Progression and Sunitinib Resistance in Renal Cell Carcinoma through Hippo Signaling Suppression
Source: Adv Sci (Weinh). 2025 Sep 15;12(44):e06367. doi: 10.1002/advs.202506367 (PMC12667553; doi:10.1002/advs.202506367)
Supplement: Supplementary file 2 — Supplemental TableS1‐S5 [file ADVS-12-e06367-s003.zip › advs71677-sup-0003-TableS2.docx]

**Supplementary Table 2.** **Targeted sequences of shRNAs used in this study.**

| Gene | Targeting sequences（5'-3'） | Source |
| --- | --- | --- |
| shTWF2-1 | GCTCCAGCAGATCCGCATTAA | HanYi Biosciences Inc. (China) |
| shTWF2-2 | CTGTGAAGGATGACCTCTCTT | HanYi Biosciences Inc. (China) |
| shYAP1-1 | GCCACCAAGCTAGATAAAGAA | HanYi Biosciences Inc. (China) |
| shYAP1-1 | CAGGTGATACTATCAACCAAA | HanYi Biosciences Inc. (China) |
| scrambled shRNA | CAACAAGATGAAGAGCACCAA | HanYi Biosciences Inc. (China) |
