# Supplementary material for: TWF2 Drives Tumor Progression and Sunitinib Resistance in Renal Cell Carcinoma through Hippo Signaling Suppression
Source: Adv Sci (Weinh). 2025 Sep 15;12(44):e06367. doi: 10.1002/advs.202506367 (PMC12667553; doi:10.1002/advs.202506367)
Supplement: Supplementary file 2 — Supplemental TableS1‐S5 [file ADVS-12-e06367-s003.zip › advs71677-sup-0006-TableS5.docx]

**Table S5** The clinicopathological characteristics of KIRC patients in TCGA cohort

| **Characteristic** | **levels** | **Overall** |
| --- | --- | --- |
| n |  | 539 |
| Gender, n (%) | Female | 186 (34.5%) |
|  | Male | 353 (65.5%) |
| Age, n (%) | <60 | 250 (46.4%) |
|  | >=60 | 289 (53.6%) |
| Pathologic stage, n (%) | Stage I | 272 (50.7%) |
|  | Stage II | 59 (11%) |
|  | Stage III | 123 (22.9%) |
|  | Stage IV | 82 (15.3%) |
| T stage, n (%) | T1 | 278 (51.6%) |
|  | T2 | 71 (13.2%) |
|  | T3 | 179 (33.2%) |
|  | T4 | 11 (2%) |
| N stage, n (%) | N0 | 241 (93.8%) |
|  | N1 | 16 (6.2%) |
| M stage, n (%) | M0 | 428 (84.6%) |
|  | M1 | 78 (15.4%) |
| OS event, n (%) | Alive | 366 (67.9%) |
|  | Dead | 173 (32.1%) |
| DSS event, n (%) | Alive | 420 (79.5%) |
|  | Dead | 108 (20.5%) |
| PFI event, n (%) | Alive | 378 (70.1%) |
|  | Dead | 161 (29.9%) |

Note: OS, overall survival; DSS, disease specific survival; PFI, progression free survival.
