# Supplementary material for: TWF2 Drives Tumor Progression and Sunitinib Resistance in Renal Cell Carcinoma through Hippo Signaling Suppression
Source: Adv Sci (Weinh). 2025 Sep 15;12(44):e06367. doi: 10.1002/advs.202506367 (PMC12667553; doi:10.1002/advs.202506367)
Supplement: Supplementary file 2 — Supplemental TableS1‐S5 [file ADVS-12-e06367-s003.zip › advs71677-sup-0002-TableS1.docx]

**Supplementary Table 1: Correlations between TWF2 expressions and clinical characteristics of ccRCC patients in the SYSU Cohort.**

| **Variable** | Total（%） | TWF2 expression | | P value |
| --- | --- | --- | --- | --- |
|  |  | Low | High |  |
| **Total**  **Gender** | 120 | 60 | 60 | 0.2663 |
| Male | 81 (67.5%) | 39 (32.5%) | 42 (35%) |  |
| Female | 39 (32.5%) | 23 (19.2%) | 16 (13.3%) |  |
| **Age (Median, range)**  ≤60  >60 | 76 (63.3%)  44 (36.7%) | 37 (30.8%)  23 (19.2%) | 39 (32.5%)  21 (17.5%) | 0.7048 |
| **TNM stage** |  |  |  | 0.0396 |
| I- II | 103 (85.8%) | 58 (48.3%) | 45 (37.5%) |  |
| III | 17 (14.2%) | 5 (4.2%) | 12 (10 %) |  |
| **WHO/ISUP Grade** |  |  |  | 0.0335 |
| I- II | 91 (75.8%) | 52 (43.3%) | 39 (32.5%) |  |
| III- IV | 29 (24.2%) | 10 (8.3%) | 19 (15.8%) |  |
