# Supplementary material for: TWF2 Drives Tumor Progression and Sunitinib Resistance in Renal Cell Carcinoma through Hippo Signaling Suppression
Source: Adv Sci (Weinh). 2025 Sep 15;12(44):e06367. doi: 10.1002/advs.202506367 (PMC12667553; doi:10.1002/advs.202506367)

Full uncropped blots

Full uncropped blots for Figure 1F

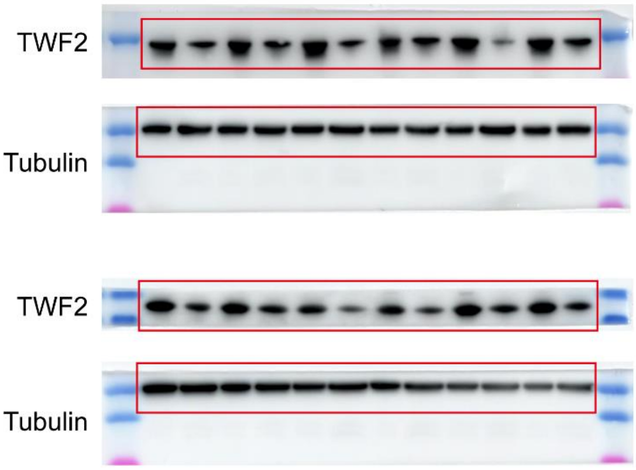

Full uncropped blots for Figure 1G

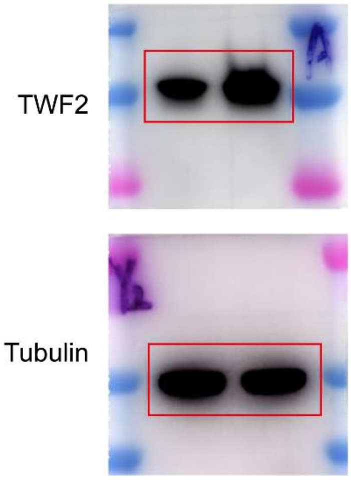

Full uncropped blots for Figure 3B

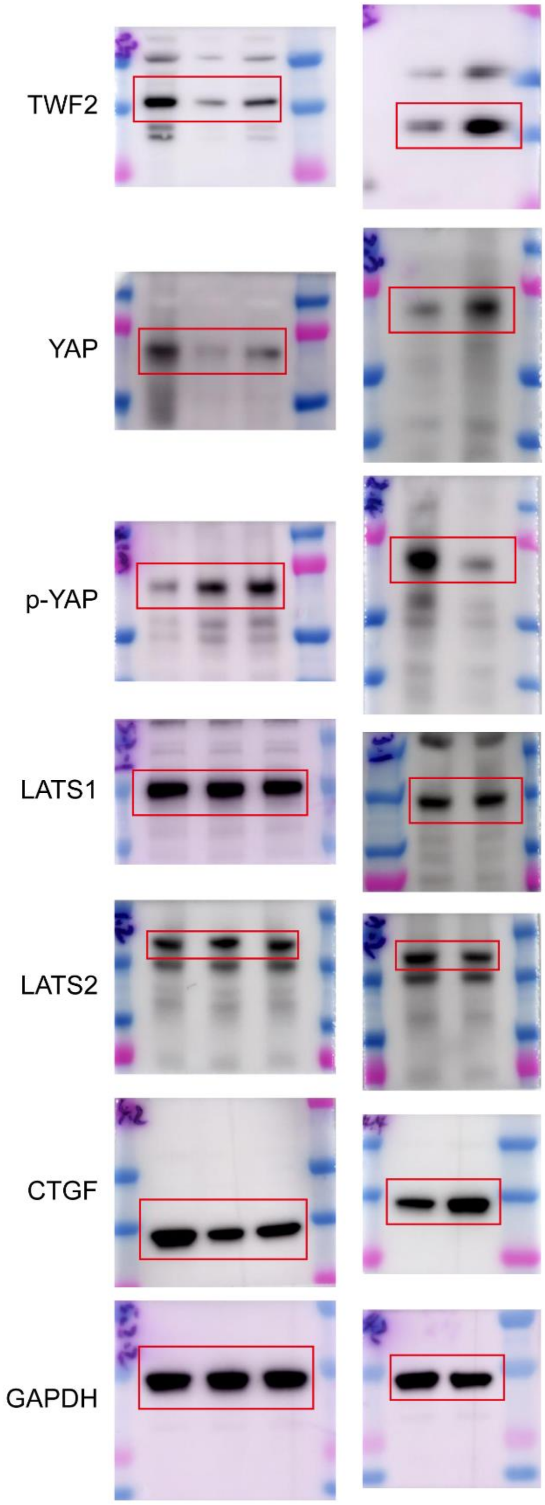

Full uncropped blots for Figure 3C

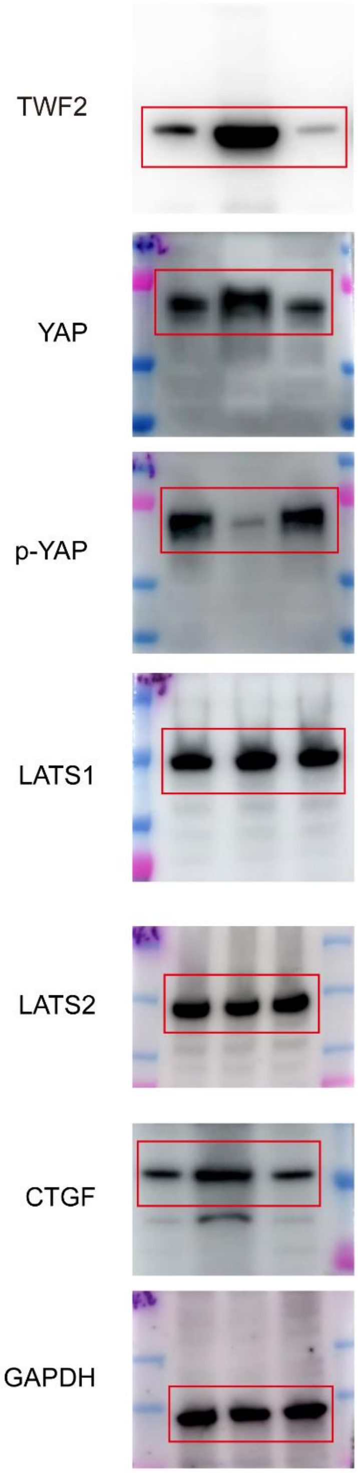

Full uncropped blots for Figure 3H

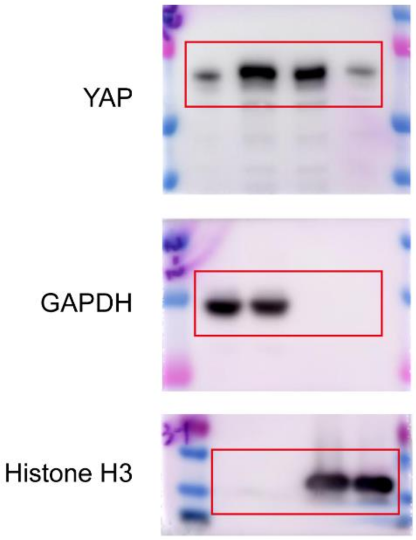

Full uncropped blots for Figure 3I

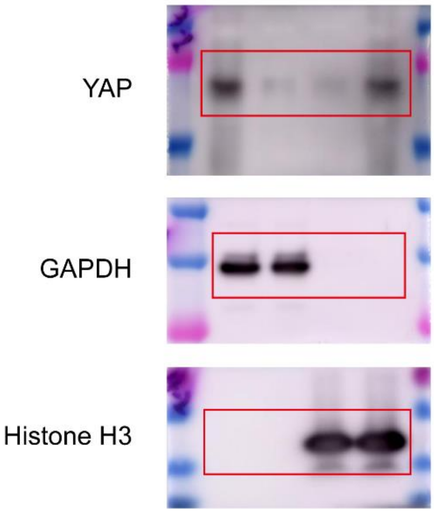

Full uncropped blots for Figure 3J

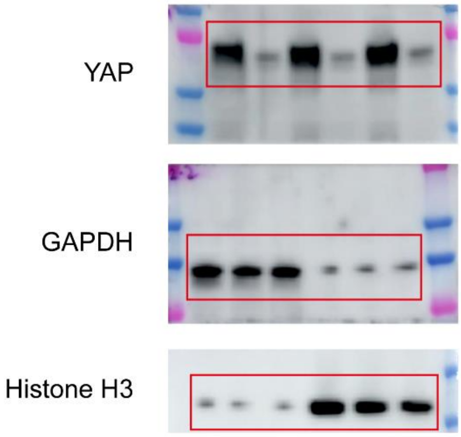

Full uncropped blots for Figure 4A

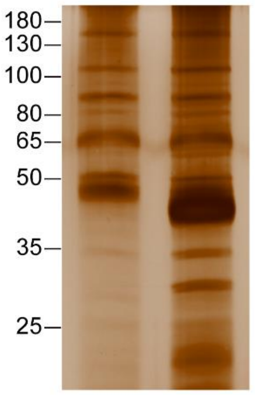

Full uncropped blots for Figure 4C

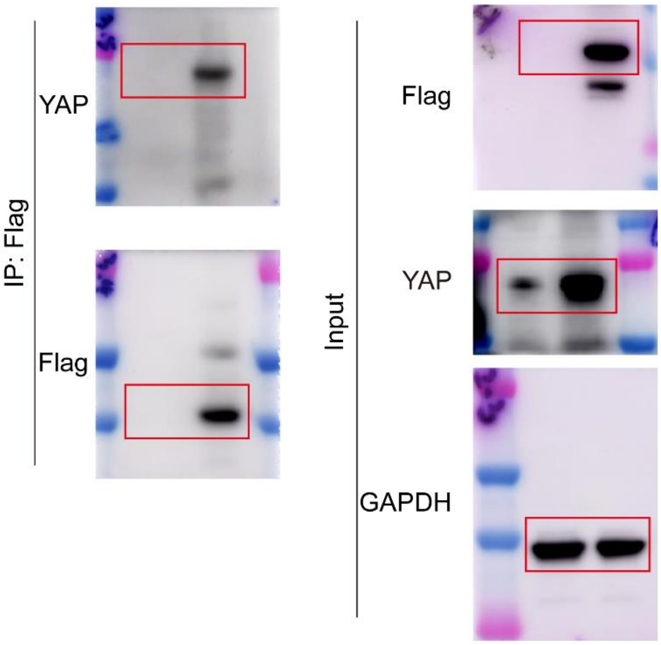

Full uncropped blots for Figure 4D

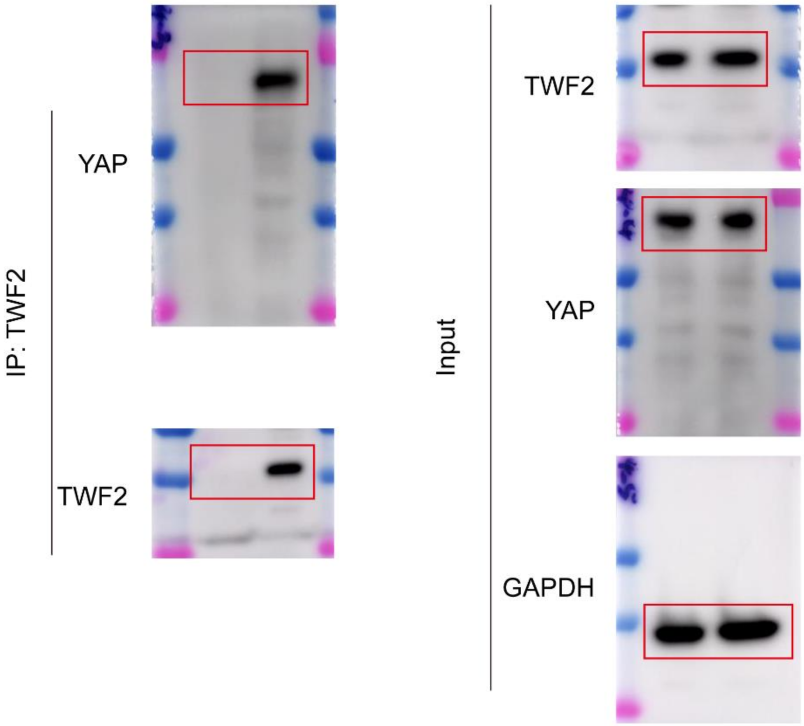

Full uncropped blots for Figure 4H

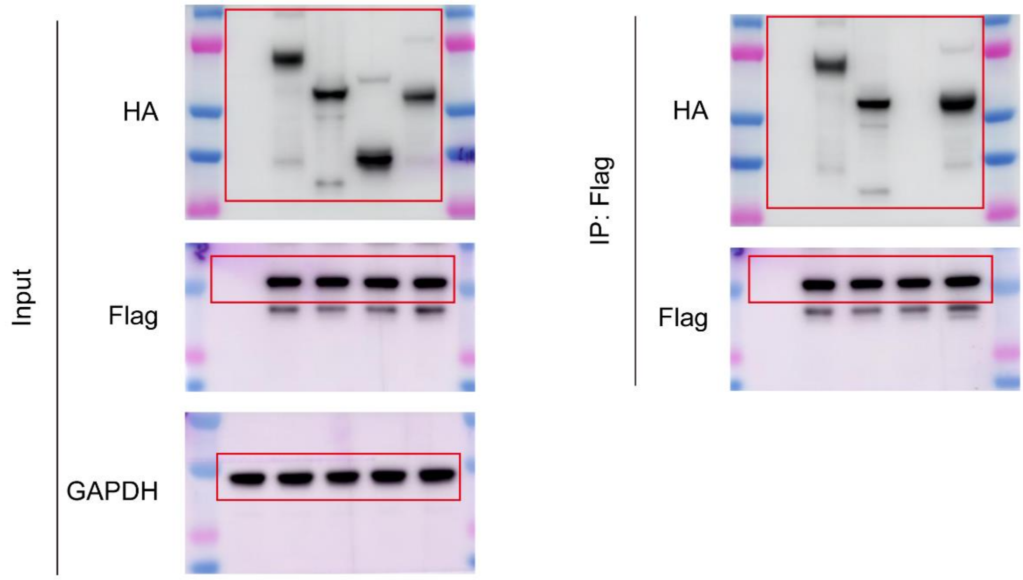

Full uncropped blots for Figure 5C

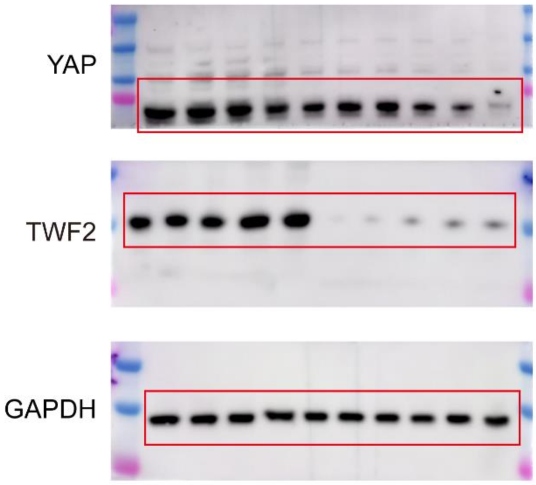

Full uncropped blots for Figure 5D

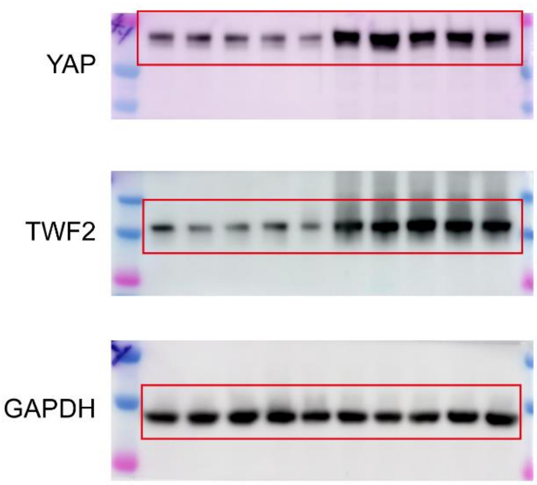

Full uncropped blots for Figure 5E

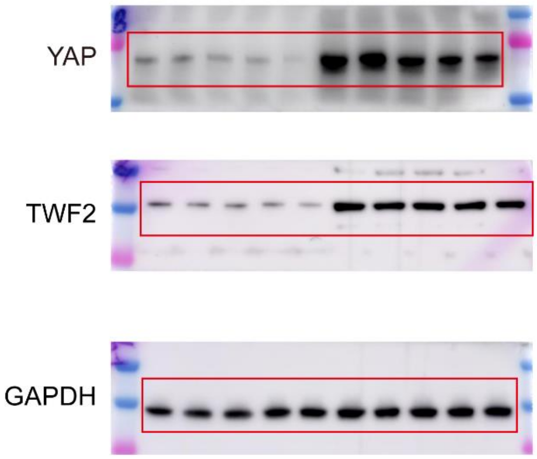

Full uncropped blots for Figure 5F

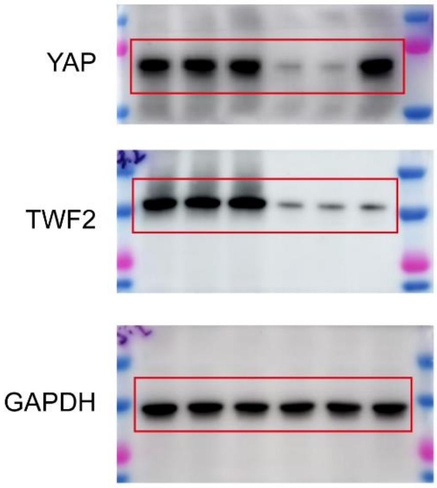

Full uncropped blots for Figure 5G

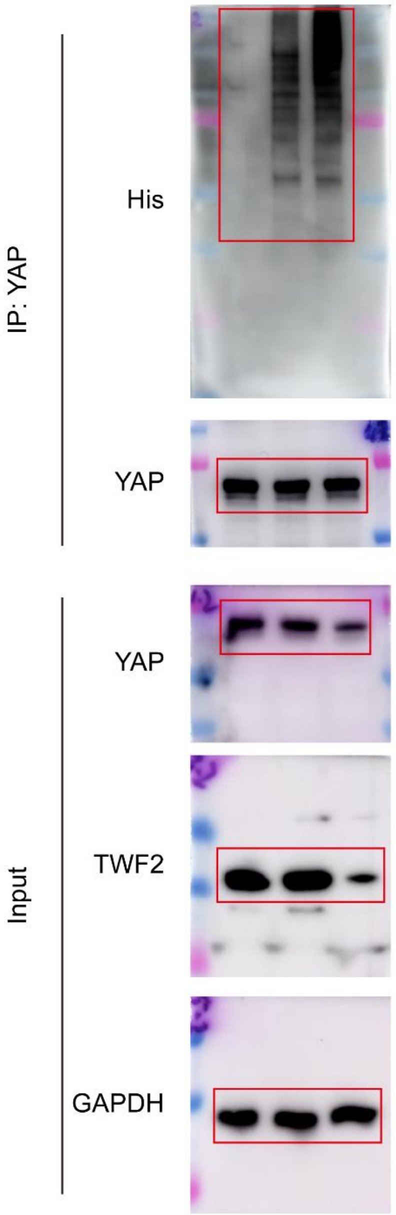

Full uncropped blots for Figure 5H

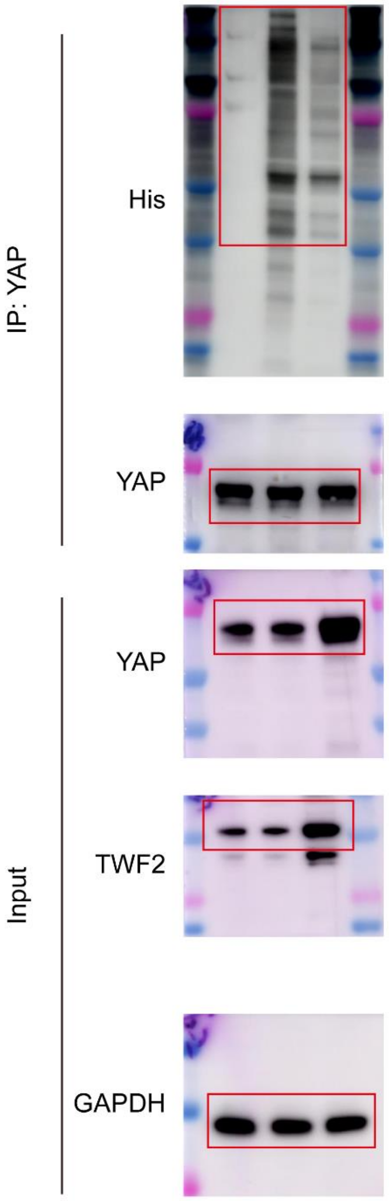

Full uncropped blots for Figure 5I

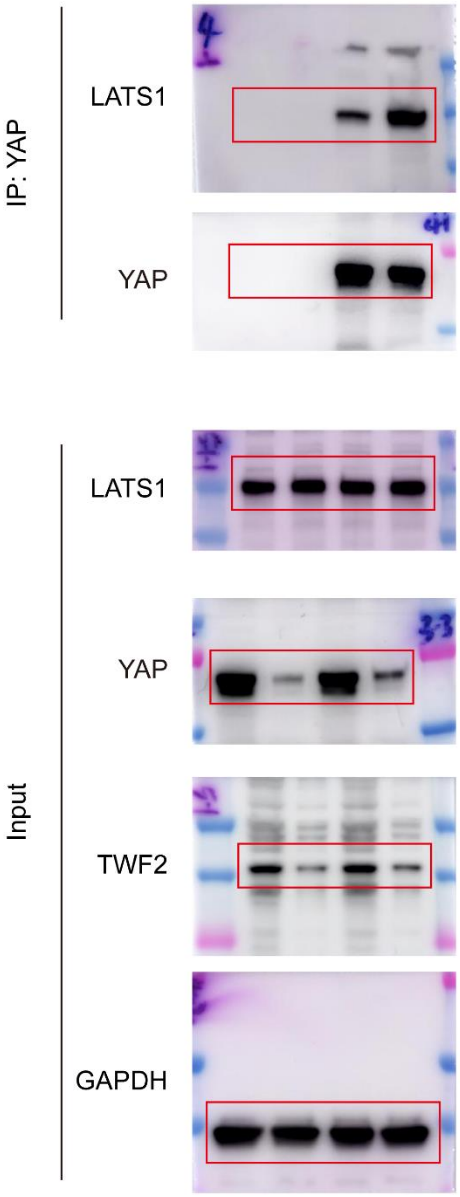

Full uncropped blots for Figure 5J

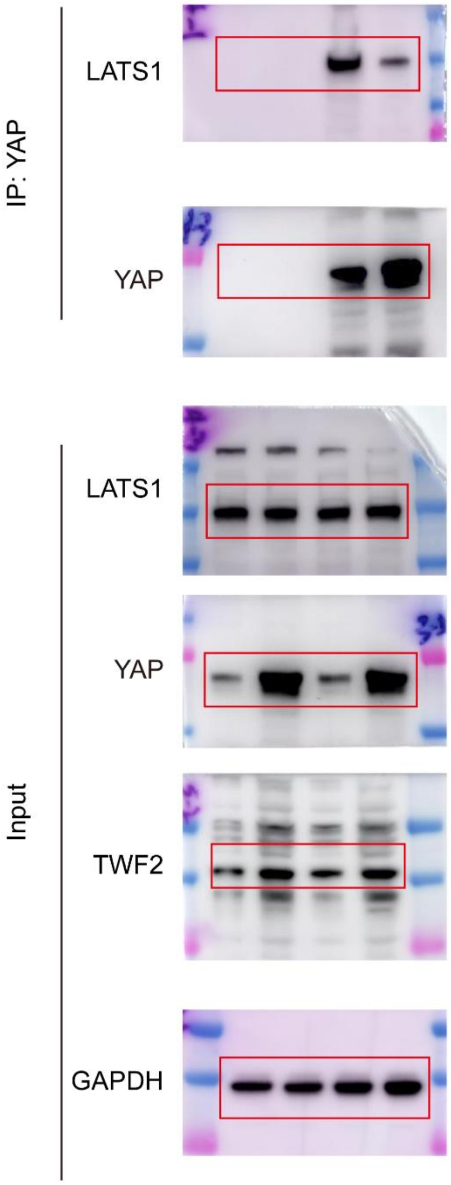

Full uncropped blots for Figure 5K

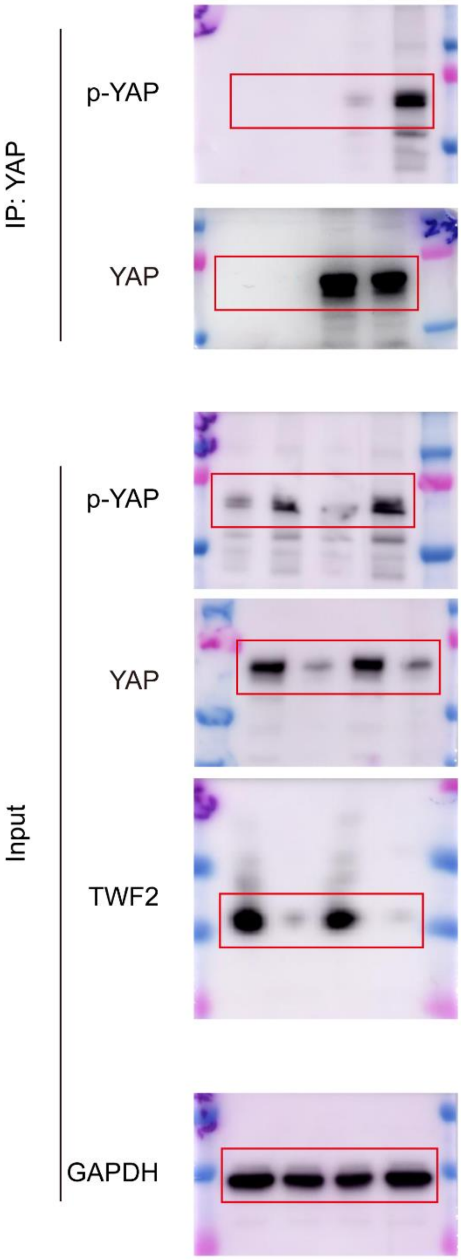

Full uncropped blots for Figure 5L

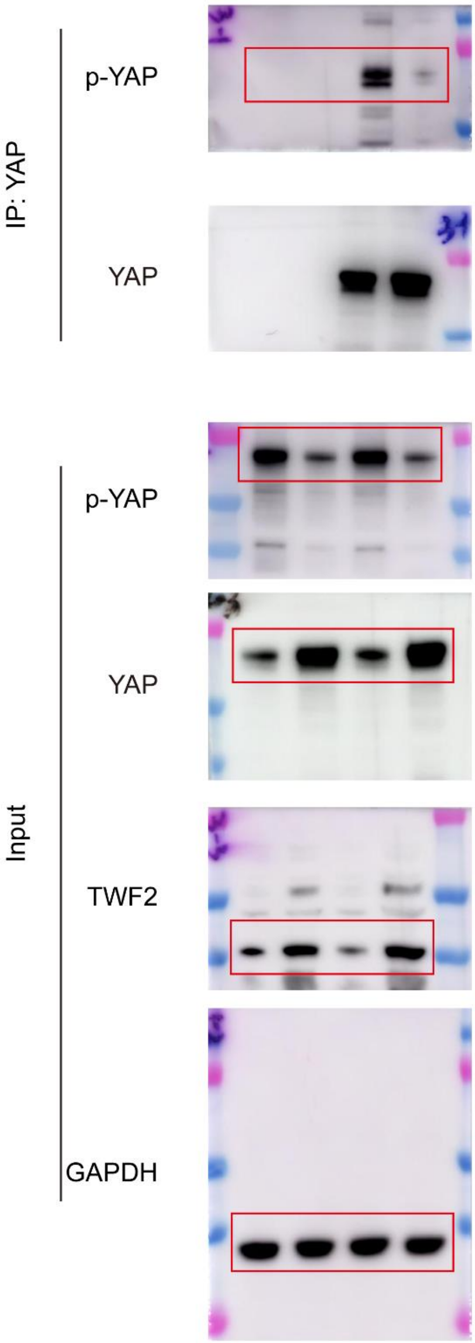

Full uncropped blots for Figure 5M

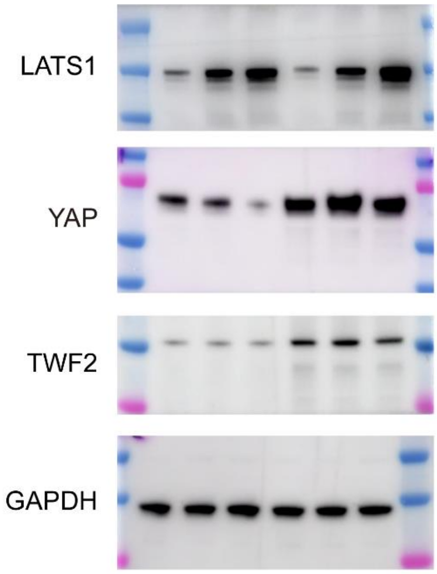

Full uncropped blots for Figure 6B

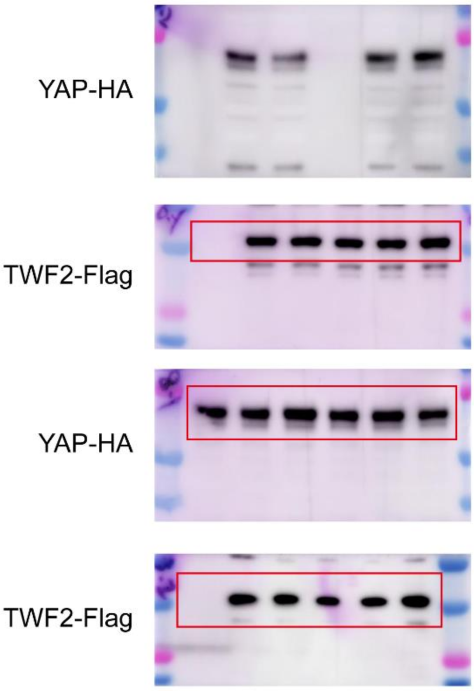

Full uncropped blots for Figure 6C

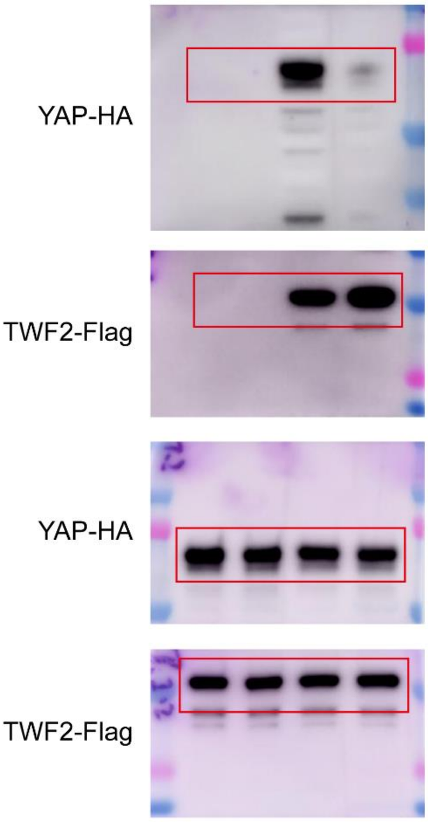

Full uncropped blots for Figure 6D

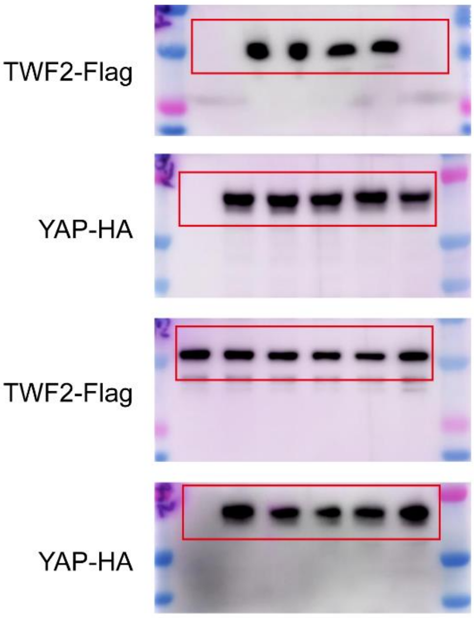

Full uncropped blots for Figure 6E

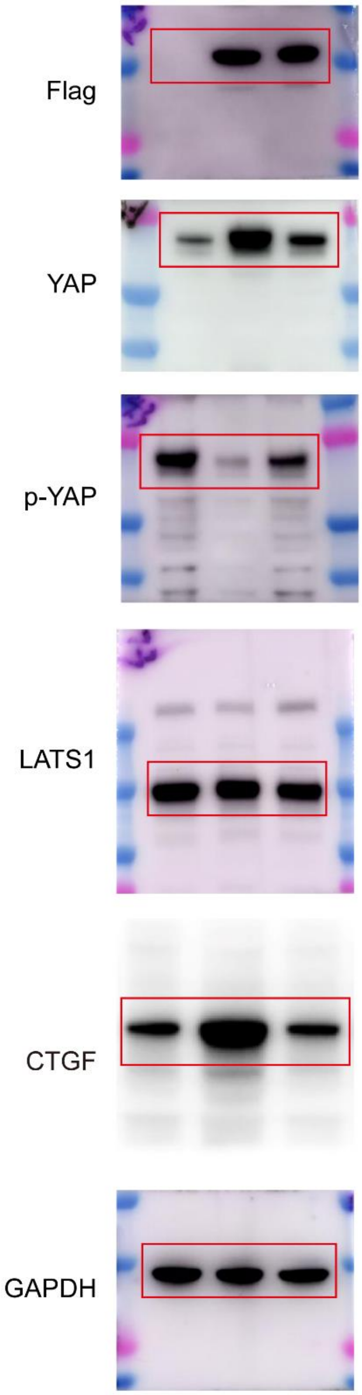

Full uncropped blots for Figure 6F

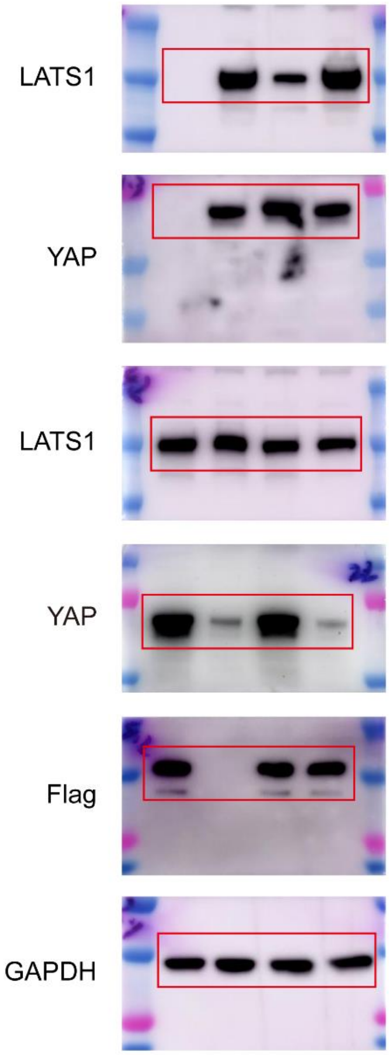

Full uncropped blots for Figure 6G

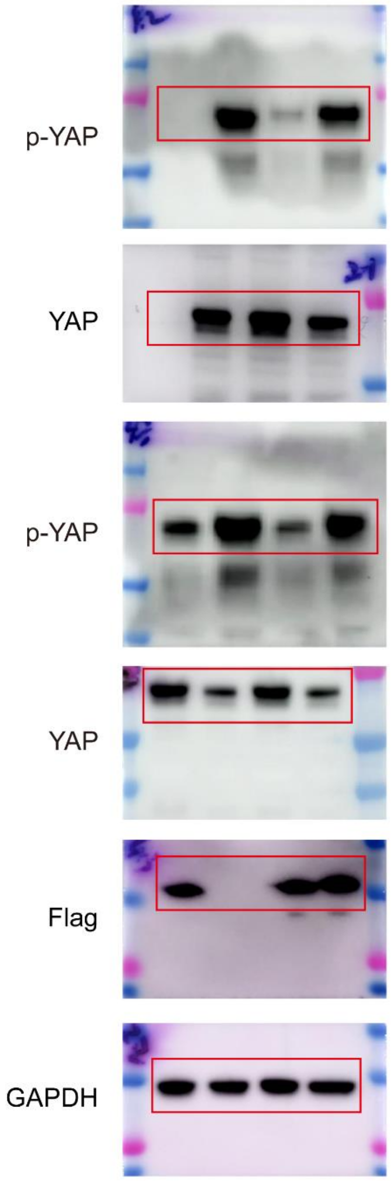

Full uncropped blots for Figure 6H

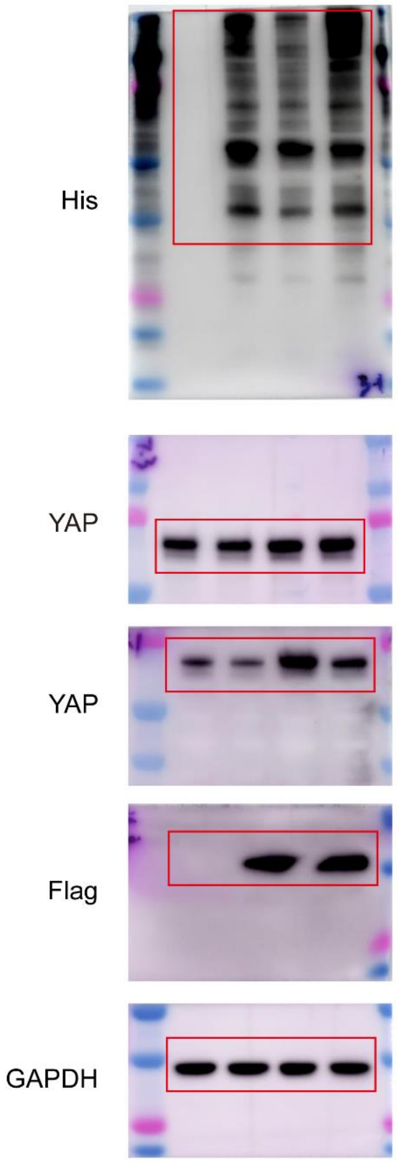

Full uncropped blots for Figure 8F

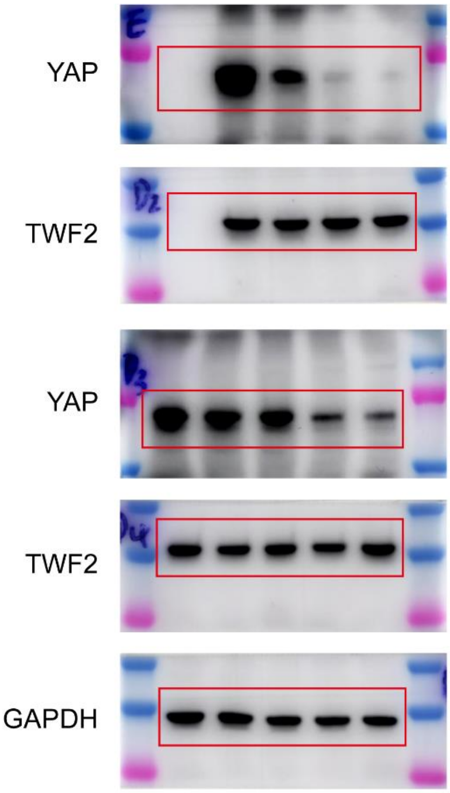

Full uncropped blots for Figure 8G

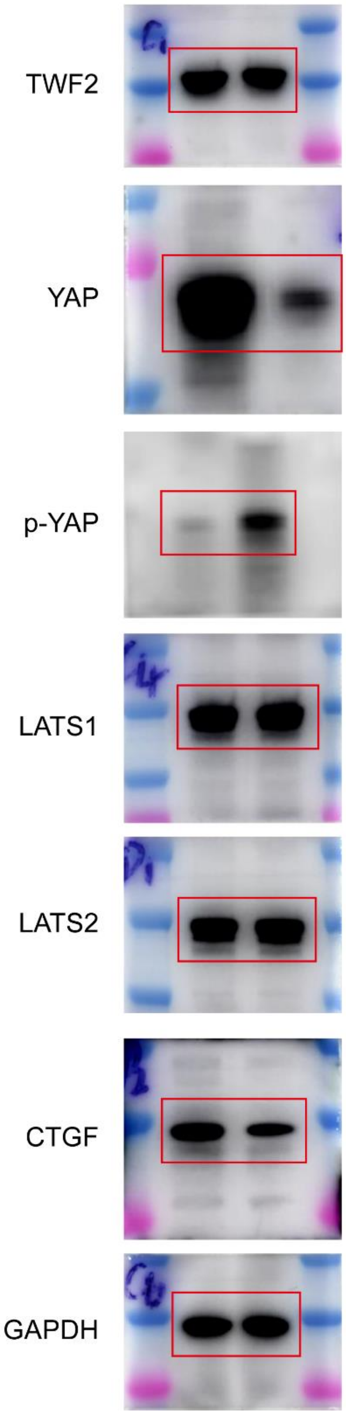

Full uncropped blots for Figure S2G

TWF2

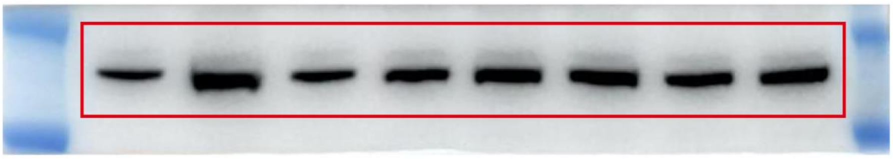

Tubulin

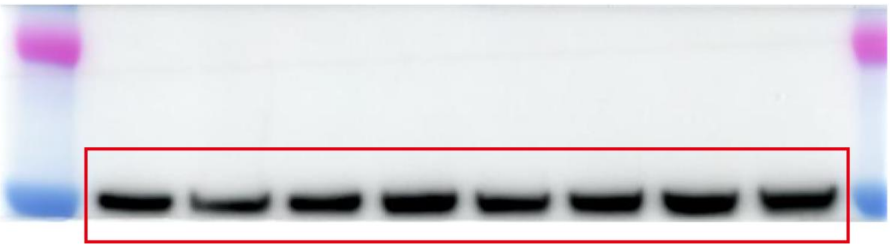

Full uncropped blots for Figure S2I

TWF2

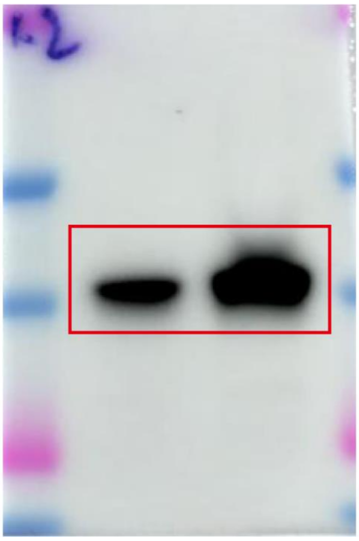

Tubulin

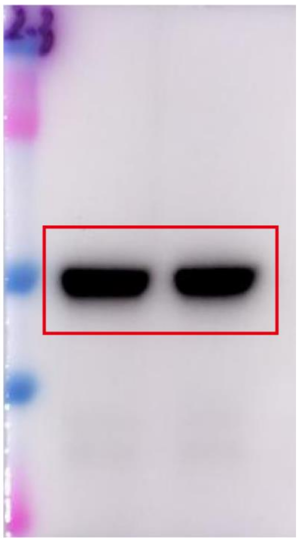

Full uncropped blots for Figure S3A

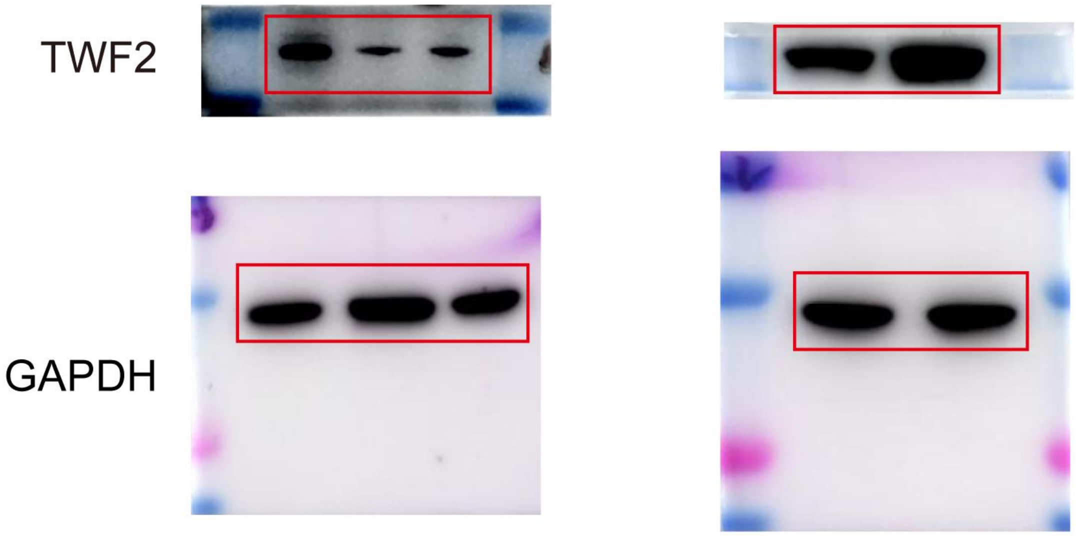

Full uncropped blots for Figure S3F

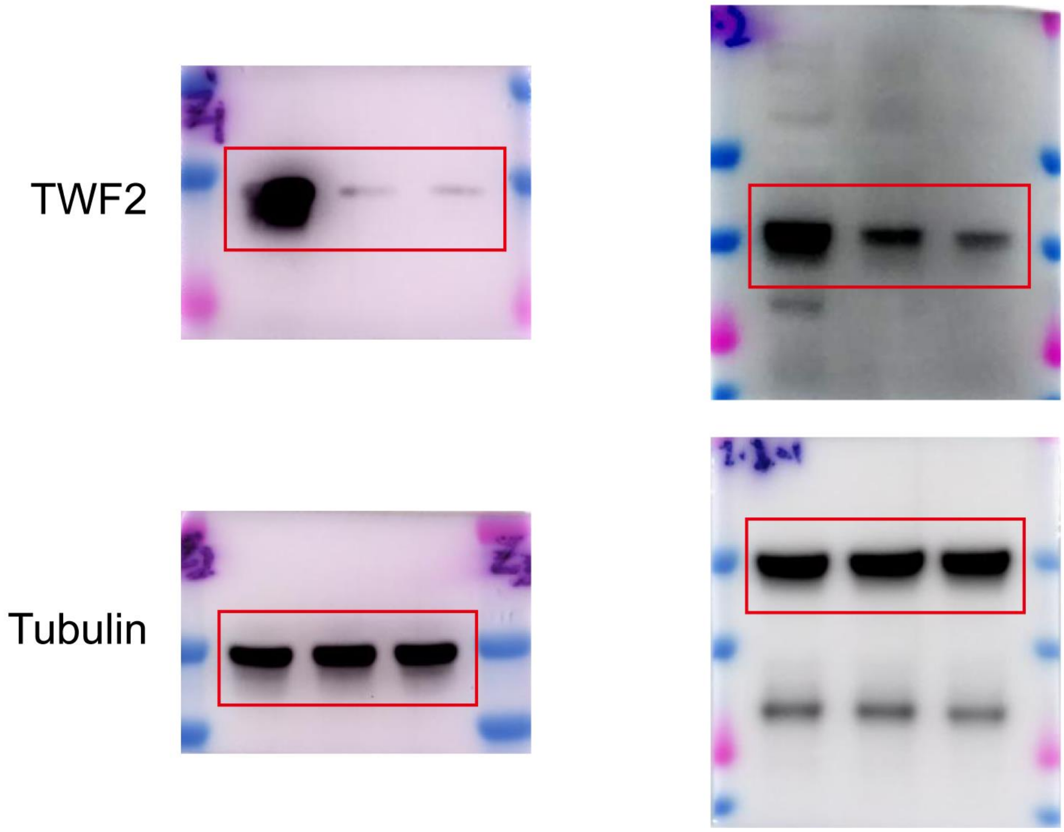

Full uncropped blots for Figure S4C

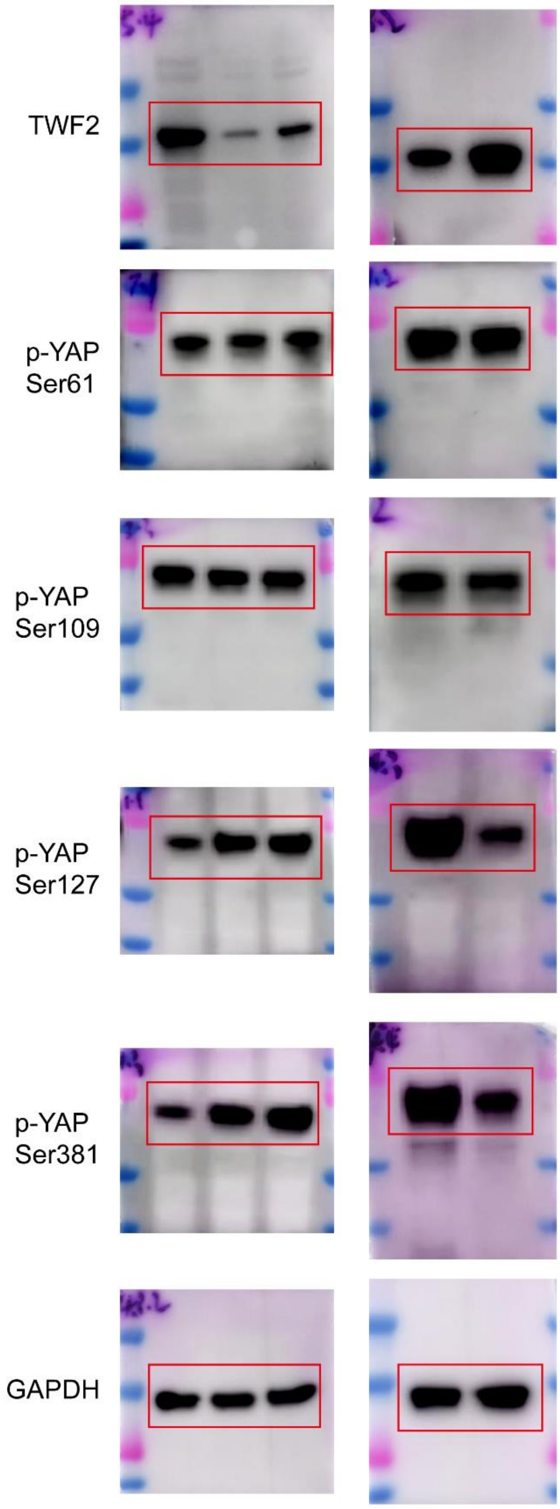

Full uncropped blots for Figure S4F

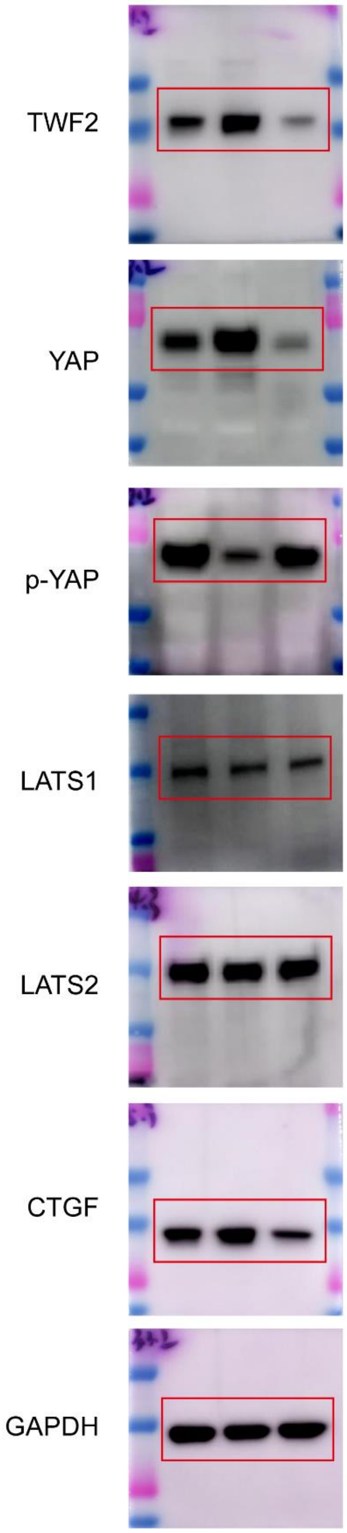

Full uncropped blots for Figure S5A

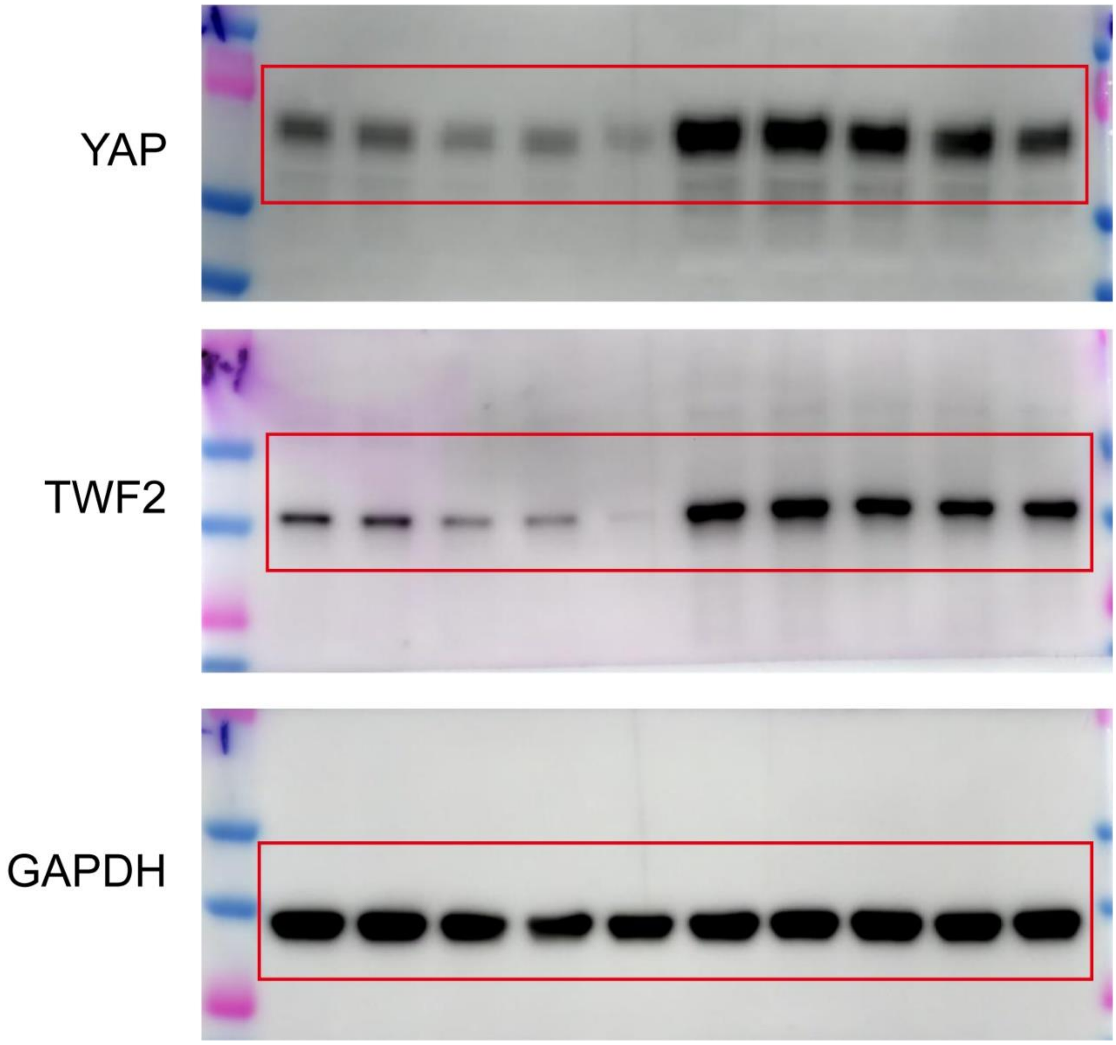

Full uncropped blots for Figure S6A

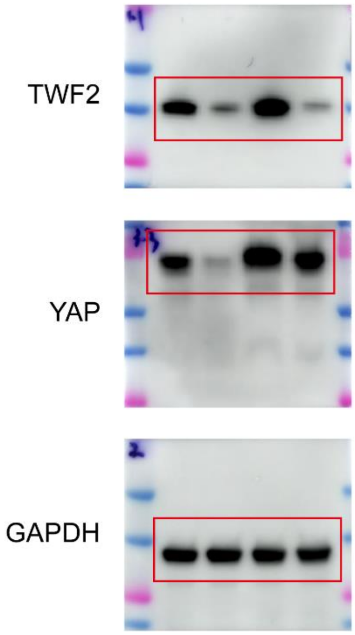

Full uncropped blots for Figure S6G

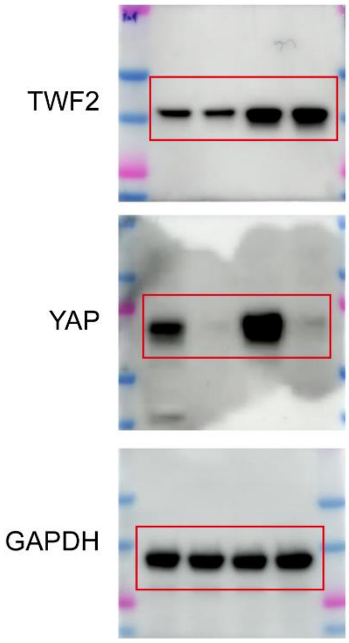

Full uncropped blots for Figure S8A

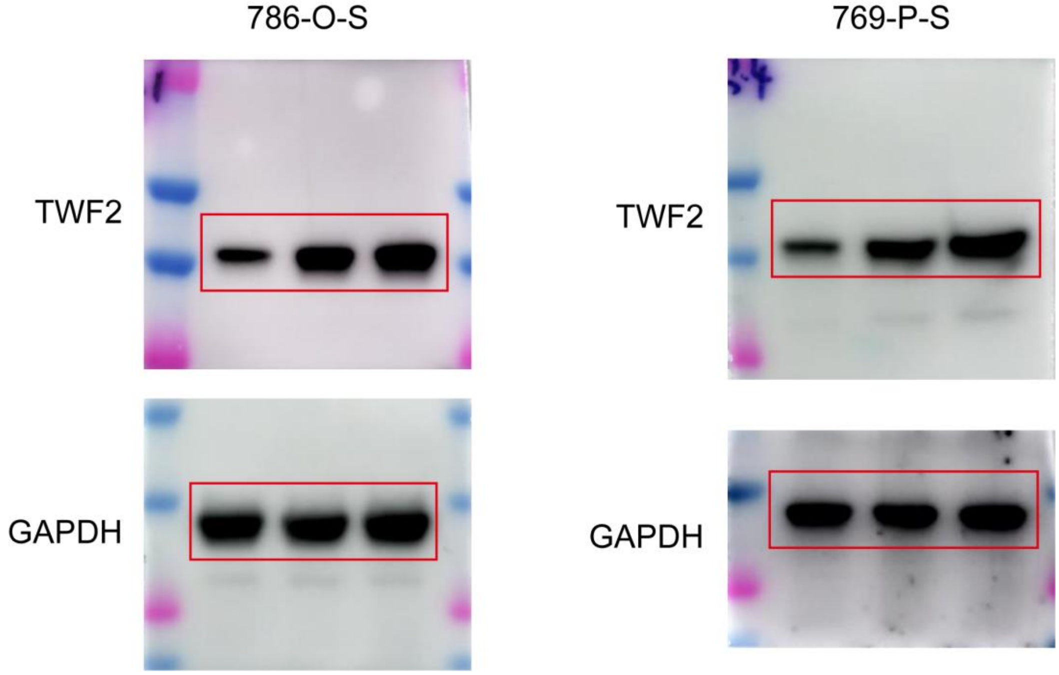

Full uncropped blots for Figure S9A

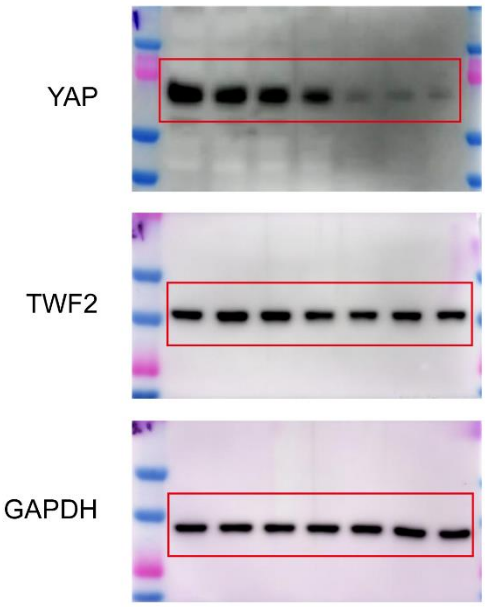

Full uncropped blots for Figure S9I

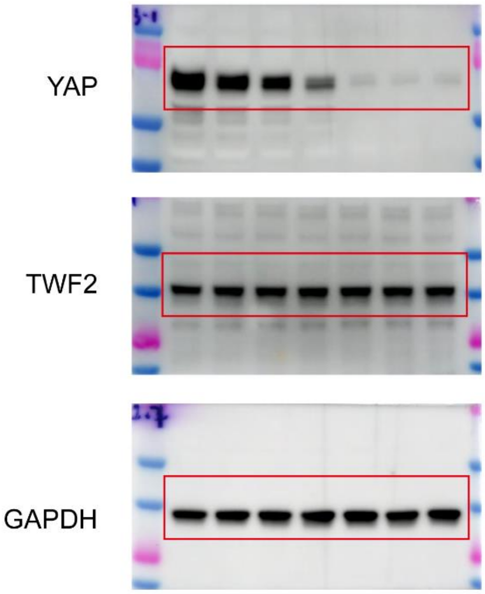

Full uncropped blots for Figure S9B

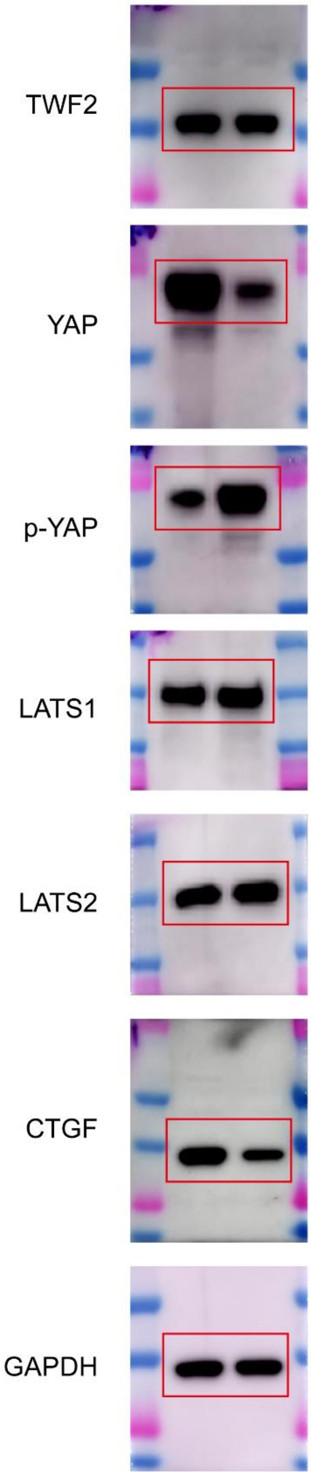

Supplement: Supplementary file 3 — Supporting Information [file ADVS-12-e06367-s001.pdf]
